# European Society of Organ Transplantation (ESOT) Consensus Statement on Prehabilitation for Solid Organ Transplantation Candidates

**DOI:** 10.3389/ti.2023.11564

**Published:** 2023-07-21

**Authors:** Coby Annema, Stefan De Smet, Ellen M. Castle, Yasna Overloop, Joost M. Klaase, Tania Janaudis-Ferreira, Sunita Mathur, Evangelia Kouidi, Maria Jose Perez Saez, Christophe Matthys, Fabienne Dobbels, Pisana Ferrari, Justyna Gołębiewska, Anna Mrzljak, Peter Girman, Michael Perch, Victor Lopez-Lopez, Colin White, Dmytro Koval, Sharlene Greenwood, Diethard Monbaliu

**Affiliations:** ^1^ Section of Nursing Science, Department of Health Sciences, University Medical Center Groningen, University of Groningen, Groningen, Netherlands; ^2^ Group Rehabilitation for Internal Disorders, Department of Rehabilitation Sciences, KU Leuven, Leuven, Belgium; ^3^ Nephrology and Renal Transplantation, Department of Microbiology, Immunology and Transplantation, KU Leuven, Leuven, Belgium; ^4^ Laboratory of Abdominal Transplantation, Department of Microbiology, Immunology and Transplantation, KU Leuven, Leuven, Belgium; ^5^ Physiotherapy, Department of Health Sciences, College of Health, Medicine and Life Sciences, Brunel University London, London, United Kingdom; ^6^ Department of Endocrinology, University Hospitals Leuven, Leuven, Belgium; ^7^ Section of Hepatobiliary Surgery & Liver Transplantation, Department of Surgery, University Medical Center Groningen, University of Groningen, Groningen, Netherlands; ^8^ Respiratory Epidemiology and Clinical Research Unit, Centre for Outcomes Research and Evaluation, Research Institute of McGill University Health Centre, Montreal, QC, Canada; ^9^ School of Physical and Occupational Therapy, McGill University, Montreal, QC, Canada; ^10^ School of Rehabilitation Therapy, Queen’s University, Kingston, ON, Canada; ^11^ Laboratory of Sports Medicine, Department of Physical Education and Sports Science, Aristotle University of Thessaloniki, Thessaloniki, Greece; ^12^ Kidney Transplant Program, Hospital del Mar, Barcelona, Spain; ^13^ Clinical and Experimental Endocrinology, Department of Chronic Diseases and Metabolism, KU Leuven, Leuven, Belgium; ^14^ Department of Public Health and Primary Care, Academic Centre for Nursing and Midwifery, KU Leuven, Leuven, Belgium; ^15^ Associazione Italiana Ipertensione Polmonare (AIPI), Bologna, Italy; ^16^ Department of Nephrology, Transplantology and Internal Medicine, Medical University of Gdańsk, Gdańsk, Poland; ^17^ Liver Transplant Center, University Hospital Center Zagreb, School of Medicine, University of Zagreb, Zagreb, Croatia; ^18^ Diabetes Center, Institute for Clinical and Experimental Medicine, Prague, Czechia; ^19^ Section of Lung Transplantation, Department of Cardiology, Rigshospitalet, Copenhagen, Denmark; ^20^ Department of Clinical Medicine, University of Copenhagen, Copenhagen, Denmark; ^21^ Department of General, Visceral and Transplant Surgery, Hospital Clínico y Universitario Virgen de La Arrixaca, Murcia, Spain; ^22^ Representative of the European Kidney Patients’ Federation, Dublin, Ireland; ^23^ Ukrainian Transplant Coordination Center, Specialized State Institution, Kiev, Ukraine; ^24^ Renal Medicine and Therapies, King’s College Hospital NHS Trust, London, United Kingdom; ^25^ Centre for Nephrology, Urology and Transplantation, Faculty of Life Sciences and Medicine, King’s College London, London, United Kingdom; ^26^ Transplantoux Foundation, Leuven, Belgium

**Keywords:** prehabilitation, solid organ transplant candidates, exercise, nutrition, psychosocial interventions

## Abstract

There is increasingly growing evidence and awareness that prehabilitation in waitlisted solid organ transplant candidates may benefit clinical transplant outcomes and improve the patient’s overall health and quality of life. Lifestyle changes, consisting of physical training, dietary management, and psychosocial interventions, aim to optimize the patient’s physical and mental health before undergoing surgery, so as to enhance their ability to overcome procedure-associated stress, reduce complications, and accelerate post-operative recovery. Clinical data are promising but few, and evidence-based recommendations are scarce. To address the need for clinical guidelines, The European Society of Organ Transplantation (ESOT) convened a dedicated Working Group “Prehabilitation in Solid Organ Transplant Candidates,” comprising experts in physical exercise, nutrition and psychosocial interventions, to review the literature on prehabilitation in this population, and develop recommendations. These were discussed and voted upon during the Consensus Conference in Prague, 13–15 November 2022. A high degree of consensus existed amongst all stakeholders including transplant recipients and their representatives. Ten recommendations were formulated that are a balanced representation of current published evidence and real-world practice. The findings and recommendations of the Working Group on Prehabilitation for solid organ transplant candidates are presented in this article.

## Introduction

Patients who need a solid organ transplant often have a compromised overall condition due to end-stage organ failure, comorbidities, deconditioning, and treatment-related adverse effects such as dialysis in end-stage kidney disease (ESKD), left ventricular assist device (LVAD) in heart failure, and oxygen therapy in end-stage pulmonary disease (ESPD) [1–3]. Although considered a frail patient population with malnutrition, low physical fitness, fatigue, and often secondary psychological challenges, it is imperative for such patients to attain, and maintain, their optimal physical and mental wellbeing, as this will help them tolerate the waiting time and the stress of transplant surgery and expedite recovery after the transplant. The time spent on the transplant waitlist provides a window of opportunity to work towards enhancing the overall condition of such patients.

Prehabilitation refers to the optimization of patient’s overall physical and psychological condition before undergoing surgery, in order to enhance his/hers ability to overcome the stress associated with the procedure, to reduce the risk of complications and to accelerate post-operative recovery, with the ultimate goal to improve survival and quality of life [4]. The approach focuses on achieving lifestyle changes and should consist of physical training, dietary management, and psychological interventions [4]. By providing a multimodal program, the complex interaction between the physical and psychological health of a patient is addressed, which is important to maximize the outcomes of the interventions [5].

Prehabilitation has shown promising results in non-transplant patients undergoing major abdominal or orthopedic procedures [6–10], with reduced overall post-operative complications and morbidity, improved aerobic capacity, and improved functional recovery and shorter length of stay. The conclusions from two systematic reviews supported the feasibility and safety of such interventions in waitlisted solid organ transplant candidates [11, 12]. In addition, observed beneficial effects included improvements in cardiorespiratory function, exercise capacity, muscular strength, mental/physical composite scores and health-related quality of life [11, 12]. There is a growing awareness and evidence that prehabilitation may not only benefit clinical transplant outcomes, but may also improve the transplant candidate’s overall health and quality of life, through adoption of a sustainable, healthy lifestyle. Despite this growing awareness and promising data, evidence-based recommendations for physical exercise, nutritional, or psychological prehabilitation interventions in candidates for solid organ transplants are not available. With regard to exercise interventions, recommendations on the role of exercise in solid organ transplantation were made by Janaudis-Ferreira et al in a position statement paper in 2019 [13].

The limited clinical guidance on how to implement prehabilitation for solid organ transplant candidates was presented as one of the priority themes at the first European Society of Organ Transplantation (ESOT) consensus conference in November 2022. Under the oversight of the ESOT guideline taskforce, and in keeping with the procedures recently established by the ESOT Consensus Platform for Organ Transplantation, leading experts presented in-depth literature evidence and proposed recommendations, which were publicly discussed and assessed by an independent jury, and consensus was formed [14]. Participants in the consensus process included not only transplant, prehabilitation and medical specialists, but also allied health professionals, patients and patient representatives.

This document presents the 2022 ESOT consensus findings and recommendations on implementing prehabilitation in the care for solid organ transplant candidates. These guidelines and recommendations undergo continual review and will be updated to reflect new evidence as it becomes available.

## Methods

The consensus development process was governed by the dedicated ESOT Guidelines Taskforce and co-organized by the ESOT sections European Liver and Intestine Transplant Association, European Kidney Transplant Association, European Pancreas and Islet Transplant Association, European Cardio Thoracic Transplant Association, European Transplant Allied Healthcare Professionals, the ESOT Education Committee and Young Professionals in Transplantation.

**FIGURE 1:**
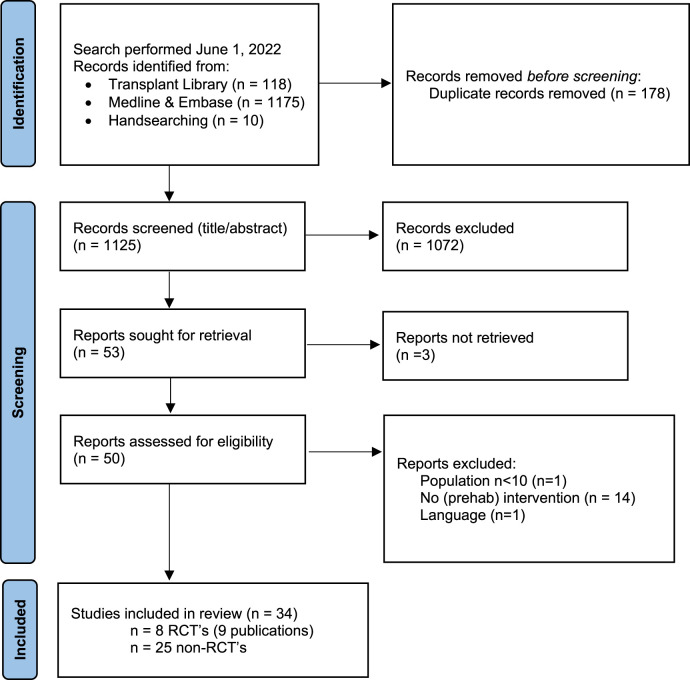
Flow chart of study selection process exercise interventions with reasons for exclusion. Note: n = number of studies. Figure adapted from: Page MJ, McKenzie JE, Bossuyt PM, Boutron I, Hoffman TC, Mulrow CD, et al. The PRIMSA 2020 statement: an updated guideline for reporting systematic reviews. BMJ 2021; 372:n71, doi:10.1136/bmj.n71. For more information visit http://www.prisma-statement.org/.

**FIGURE 2:**
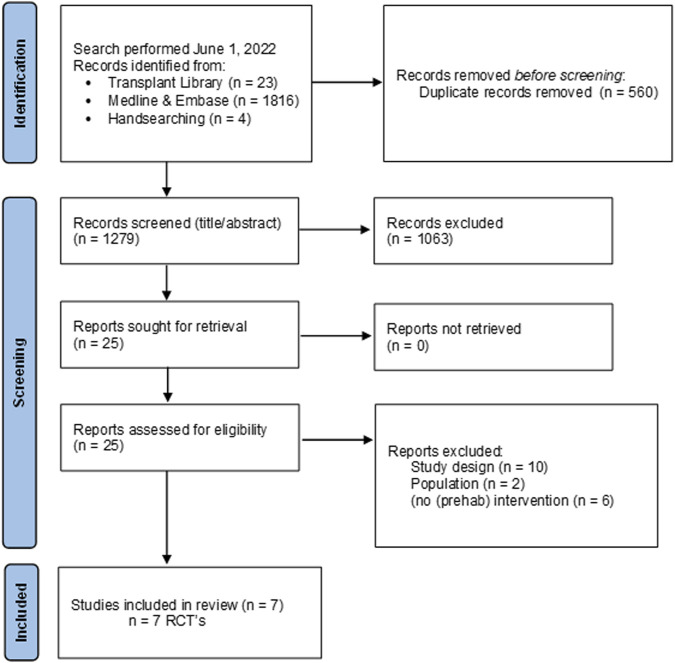
Flow chart of study selection process nutritional interventions with reasons for exclusion.

**FIGURE 3:**
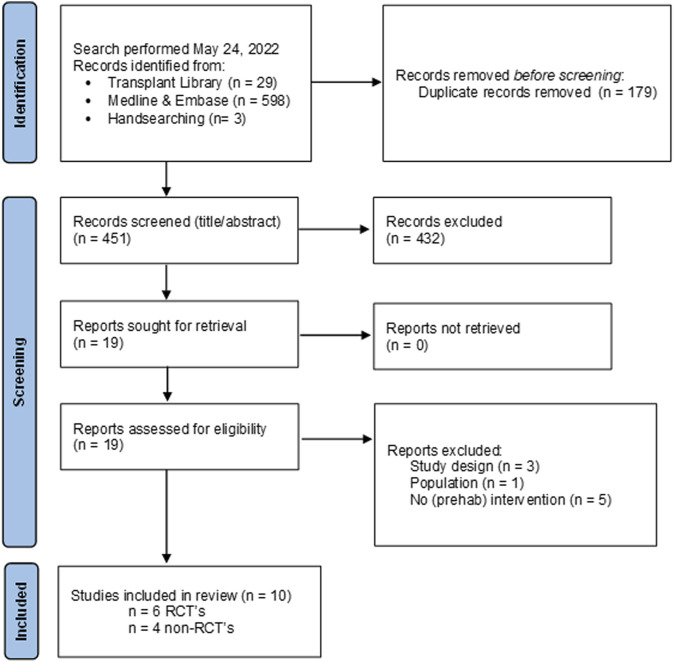
Flow chart of study selection process psychosocial interventions with reasons for exclusion.

**FIGURE 4:**
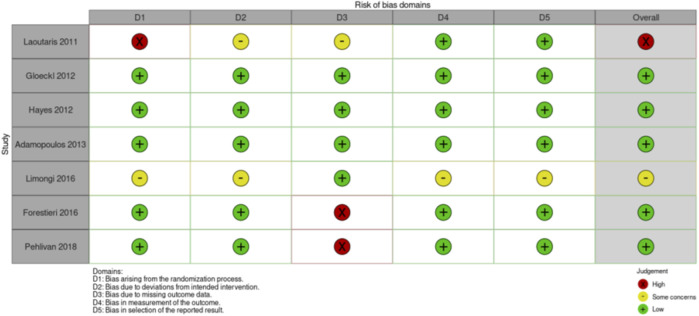
Risk of Bias assessment RCTs exercise intervention studies.

**FIGURE 5:**
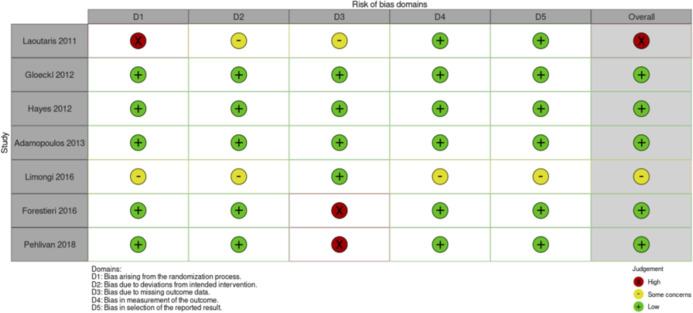
Risk of Bias assessment RCTs nutritional intervention studies.

**FIGURE 6:**
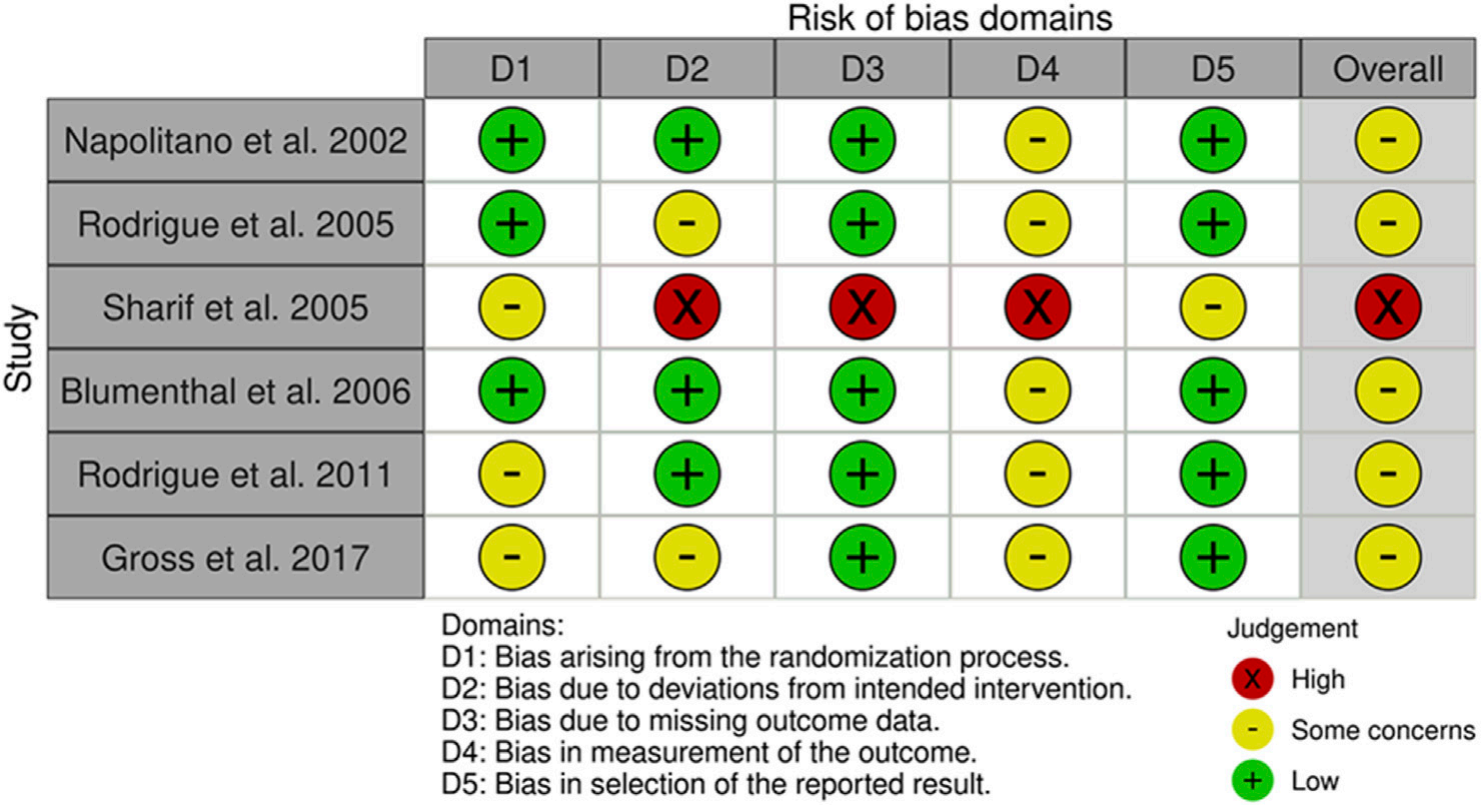
Risk of Bias assessment RCTs psychosocial intervention studies.

The consensus development process followed the methodology stipulated by the ESOT Consensus Platform as recently published in detail [14]. In brief, the subsequent steps were as follows:

i) Prehabilitation for solid organ transplant candidates was selected as a priority topic for the first ESOT Consensus Conference, as published [14].
ii) A specific steering committee was selected, consisting of experts in the topic field, members from the Centre for Evidence in Transplantation, a Young Professional in Transplantation representative, and a guideline taskforce member to liaise with ESOT.
iii) The steering committee identified key relevant questions related to prehabilitation of solid organ transplant candidates (heart, lung, liver, kidney) using to the Population, Intervention, Comparator and Outcome (PICO) methodology [15] (Table 1).
iv) The staff of the Centre for Evidence in Transplantation performed systematic literature reviews that were informed by the PICO questions and thus related to exercise, nutritional and psychological interventions in solid organ transplant candidates. The search strategy is presented in Supplementary Table S1. The PRISMA diagrams from the evidence review are shown in Figures 1–3. As the number of publications was expected to be limited, selection was not limited to randomized clinical trials but also included studies that used a pre/post or case-control design, prospective and retrospective studies (cohorts or registry), feasibility studies and pilot studies. Reviews and meta-analyses were included for hand searching of bibliographies for additional literature. Studies were included only if a minimum of 80% of study participants were formally waitlisted for a solid organ transplant. Case reports on fewer than 10 patients, conference abstracts, and letters to the editor were excluded, as was non-English literature. The literature evidence relating to the PICO questions was summarized, as shown in Tables 2–4 and Supplementary File S1.
v) The steering committee integrated the literature evidence and formulated recommendations (Supplementary File S1). When proposing recommendations for each question, the quality of evidence was considered as evaluated by the GRADE approach [16]. This included risk of bias (Figures 4–6), which was assessed by two independent reviewers, and an additional third one if disagreement occurred. The strength of the individual recommendations was rated as strong or weak.
vi) Jury members, who were not part of the steering committee were selected and vetted by the guideline taskforce and were comprised of allied health professionals, patients (representatives), transplant physicians, and transplant surgeons.
vii) Consensus was generated using discussion within the entire working group and modified Delphi methodology including consensus polling, followed by jury voting of the recommendations during a session at the ESOT Consensus conference in Prague [17].
viii) A committee of validating experts validated the recommendations using the AGREE II guidelines [18].

**TABLE 1:** PICO questions and criteria for analysis.

1	In adult candidates for lung, liver, kidney and heart transplantation: What is the evidence for the effectiveness of pre-transplant exercise training, nutritional support and psychosocial interventions, as measured by the criteria prehabilitation efficacy outcomes, clinical outcomes and patient-reported outcomes
	Criteria for analysis:
	Effectiveness of the prehabilitation program: Maximal exercise capacity, Functional exercise capacity, Muscle strength, Nutritional status, Body composition, BMI, Cardio metabolic risk profile, Distress (anxiety/depression), Fatigue, Frailty
	Clinical outcomes: Mortality (pre-/post-transplant); Hospital (re-)admissions (pre-/post-transplant); Length of hospital stay (pre-/post-transplant); Complications after transplant surgery; Graft survival; Rejection episodes
	Patient reported outcomes: Health related Quality of Life (HRQoL), Activities of daily living
2	In adult candidates for lung, liver, kidney, and heart transplantation: What is the evidence for the type of pre-transplant exercise, nutritional support and psychosocial interventions?
3	In adult candidates for lung, liver, kidney, and heart transplant candidates: Which relevant outcomes need to be measured to evaluate the effect of the pre-transplant exercise and physical therapy, nutritional support and psychosocial interventions?
4	In adult candidates for lung, liver, kidney and heart transplantation: What is the evidence for the feasibility of prehabilitation, as measured by the criteria enrolment, retention, acceptability, fidelity, safety?
	Criteria for analysis:
	Enrolment: the number of screened patients who met the eligibility criteria (n/%), the number of eligible patients who were recruited for the study (n/%)
	Retention: the number of participants that were retained in the intervention study, drop-out rate (n/%), reasons for drop-out
	Acceptability: the perception among professionals and participants that the intervention is agreeable, appropriate, or satisfactory
	Fidelity: the degree to which the intervention was implemented as it was intended, as measured by adherence to the program protocol by the interventionist and participants, Safety: occurrence of adverse events

**TABLE 2:** Summary of RCTs- Exercise interventions.

	First author, year (country of origin)	Sample characteristics Tx-type, total N, n per group, % male, age (y) (mean (sd) or median (range))	Intervention(s) and measurement points	Effectiveness outcomes	Results – Effectiveness outcomes ↑ = significant increase ↓ = significant decrease ≈ no difference	Results – Feasibility outcomes
1	Laoutaris, 2011 (Greece)	HTx candidates with LVAT or BiVAT n = 21	All participants were advised to walk every day for 30–45 min.	VO_2_peak	↑ within I; I ≈ C	Enrolment: 21/23 (91%) of eligible patients
		I n = 14 100% male Age 37 (±18)	I: 10 week, homebased aerobic exercise (45 min, 3–5x/week, intensity y12–14/20 RPE) and hospital-based IMT (until exhaustion, 2–3x/week, intensity 60% MIP)	6MWT	↑ within I; I ≈ C	Attrition: 15/21 (71%) completed (I 10/14 (71%); C 5/7 (71%)), all drop out due to Tx
		C n = 7 80% male Age 42 (±15)	C: Usual care	QOL (MLwHFQ)	↑ within I; I ≈ C	Fidelity (participants): NR
			Assessments: - Pre-intervention - Post-intervention	PIMax and sustained PImax	↑ within I; I ≈ C	Fidelity (interventionist): NR
				Lung volumes (inspiratory capacity)	↑ within I; I ≈ C	Acceptability: patients enjoyed training and seemed more enthusiastic compared with patients in the control group
				Dyspnea after 6MWT	I ≈ C	Safety: no adverse events occurred during the training period
2	Gloeckl, 2012 (Germany)	LuTx candidates following COPD stage IV diagnosis n = 71	All participants received strength training (four to six exercises, 3 sets of 30 repetitions, at maximal tolerated load), breathing therapy, education, and psychological support.	6MWT	≈ increase in I andC	Enrolment: 71/97 of eligible patients
		I n = 36 49% male Age 52 (±6)	I: 3-weeks, hospital-based high-intensity interval training, 10–36 min per session, 5–6x/week, 1–2 sessions/day, intensity repeated bouts of 30 s at 100% Wmax alternated by 30 s rest	QOL (SF-36 PCS and MCS)	PCS: ≈ within I; ↑ within C; I ≈ C MCS: ↑ within I; ≈ within C; I ≈ C	Attrition: 60/71 completed (I 30/36 (83%); C 30/35 (86%)). Dropout due to: I acute exacerbation (n = 4), non-compliance with study protocol (n = 1), other (n = 1); C acute exacerbation (n = 3), Tx (n = 1), other (n = 1)
		C n = 35 44% male Age 55 (±7)	C: 3-weeks, hospital-based, moderate-intensity aerobic exercise, 10–30 min/session, 5–6x/week, 1-2 sessions/day, intensity 60% Wmax	W_max_	≈ increase in I and C	Fidelity (participants): no difference in number of exercise sessions or total work performed per group. I: 14.9 (±1.9); C: 14.7 (±1.5)
			Assessments: - Pre-intervention - Post-intervention	During exercise - SpO_2_ - TCPCO_2_ - Dyspnea - leg fatigue	I ≈ C I ≈ C ↓ in I I ≈ C	Fidelity (interventionist) NR
				Unintended breaks (number and time) during exercise	↓ number and ↓ duration in I	Acceptability: NR
				PaO_2_ and PaCO_2_	I ≈ C	Safety: no serious adverse events occurred
				lung function (DLCO, FEV1, FEV1/IVC)	I ≈ C	
3	Hayes, 2012 (Australia)	HTx candidates with LVAT n = 14	All participants followed a progressive walking program: They were advised to walk a minimum of 5 days per week at 13 RPE and increased their walk progressively up to 60 min.	VO_2_peak	↑ in; I ≈ C	Enrolment: 14/18 (78%) of eligible patients
		I n = 7 86% male Age 48.7 (±14.5)	I: 8-week, gym-based aerobic and strength training (60 min/session, 3x/week, intensity cycling at 50% VO_2_reserve; treadmill at 60% of the speed averaged during the 6MWT; strength: three upper limb and three lower limb exercises using weight machines and free weights, 2 sets of 10 reps).	6MWT	↑ in I and C; I ≈ C	Attrition: 14/14 (100%) completed
		C n = 7 86% male Age 45.9 (±14.6)	C: Usual care, which included a walking program.	QOL (SF36)	↑ within I; I ≈ C	Fidelity (participants): I: participation in 21.3 ± 1.5 of possible 24 sessions. Reasons for missed sessions: conflicting medical appointment (79%) and conflicting family demands (21%). C: 100% compliance to the walking program
			Assessments: - Pre-intervention - Post-intervention	W_max_	↑ in I and C	Fidelity (interventionist): NR
						Acceptability: NR
						Safety: no adverse events occurred
4	Adamopoulos, 2013 (Greece)	HTx candidates with LVAT or BiVAT n = 22	All participants were advised to walk every day for 30–45 min.	Thyroid hormone signalling (TRα1, p/t-AKT and p/t-JNK)	↑ within I; I ↑ C	Enrolment: 22/26 (85%) of eligible patients
		I n = 11 91% male Age 39.7 (±4.3)	I: 12 weeks, aerobic training (home-based, 45 min/session, 4x/week, intensity 12-14/20 RPE) and IMT (hospital-based, until exhaustion, 3x/week, intensity 60% PImax	VO_2_peak	↑ within I; I ↑ C	Attrition: 22/22(100%) completed
		C n = 11 82% male Age 40.9 (±4.9)	C: Usual care	NT-proBNP (marker of heart failure)	↓ within I; I ↓ C	Fidelity (participants): NR
			Assessments: - Pre-intervention - Post-intervention			Fidelity (interventionist): NR
						Acceptability: NR
						Safety: NR
5	Limongi, 2014 and 2016 (Brazil)	LiTx candidates n = 49		MIP	↑ in I and C	Enrolment: 49/49 (100%) of eligible patients
		I n = 22 79% male Age 55.8 (±5.4)	I: 3-months, home-based, daily exercises illustrated in a manual (3 × 15 repetitions of diaphragmatic breathing exercises, diaphragmatic isometric exercise, Threshold IMT®, lifting upper limbs with a bat and strengthening the abdomen). Duration training sessions varied by patient. Intensity reported only for diaphragmatic breathing (1 kg on the belly). Supervision once a month at distance.	MEP	↑ in I and C	Attrition: 37/49 (76% completed; I 14/22 (64%); C 23/27 (85%)). Dropouts due to I: LiTx (n = 2), death (n = 3), declined to perform exercise (n = 3), C LiTx (n = 1), death (n = 3)
		C n = 27 78% male Age 55.4 (±9.9)	C: Usual care	Spirometry (FVC, FEV1)	no changes	Fidelity (participants): NR
			Assessments: - Pre-intervention - Post-intervention	QOL (SF-36)	↑ in I and C on general health and mental health subscale; ↑ within I on functional capacity, not in C, but without between group differences	Fidelity (interventionist): NR
				Surface EMG of diaphragm	I ↓ C	Acceptability: NR
				Surface EMG of rectus abdominis	no changes	Safety: NR
				Ascites presence	I ≈ C	
6	Forestieri, 2016 (Brazil)	HTx candidates n = 24		6MWT	↑ within I; I ↑ C	Enrolment: 24/27 (89%) of eligible patients
		I n = 12 71% male Age 48.3 (±10.2)	I: ∼22 days, hospital-based, intermittent aerobic (stationary cycle ergometer exercise: 5 periods of 3 min cycling and 1 min res, 20 min/session, 2x/day, intensity 3–4/10 RPE	MIP	↑ within I; I ↑ C	Attrition: 18/24 (75%) completed (I 7/12 (58%); C 11/12 (92%)). Dropouts due to: I: incapacity to complete the stationary cycle ergometer exercise (n = 5); C: acute severe arrhythmias (n = 1)
		C n = 12 82% male Age 48.0 (±11.2)	C: ∼19 days, hospital-based, breathing exercises and global active exercises of the upper and lower limbs in the upright seated position (2x/day, intensity: 3–4/10 RPE)	FVC	NR	Fidelity (participants): 42% lost for follow-up
			Assessments: - Pre-intervention - Post-intervention	FEV1	NR	Fidelity (interventionist): NR
				NT-proBNP	NR	Acceptability: 42% were incapable to complete the intervention program
						Safety: NR
7	Pehlivan, 2018 (Turkey)	LuTx candidates n = 34	All participants participated in a home-based pulmonary rehabilitation program: breathing exercises (local expansion exercises, diaphragmatic breathing, and pursed lip breathing), free walking, and upper and lower body strengthening with resistance bands. Participants completed a weekly chart that was reviewed by the physiotherapist.	MIP	↑ within I; I ↑ C	Enrolment: 34/38 (89%) of eligible patients
		I n = 17 59% male Age 36.1 (±15.9)	I: 3-months, 5x/week (supervised 2x/week; home-based 3x/week) standard pulmonary rehabilitation (aerobic exercises: treadmill, cycle and arm ergometer, 15 min per exercise modality/ session, intensity: 50%–70% of HRmax and resistance exercises: dumbbell and free weight bags, 8–12 reps, one to two sets/ session, intensity 20%–40% 1-RM) + IMT with Powerbreathe device (15 min/session, 2x/day, 5 days/week, intensity initial 30% of MIP, progressed to 60% MIP)	MEP	↑ in I and C	Attrition: 34/34 (100%) completed
		C n = 17 65% male Age 39.0 (±12.4)	C: Usual care, including standard pulmonary rehabilitation (see above)	6MWT	↑ in I and C, but greater in I than C	Fidelity (participants): NR
			Assessments: - Pre-intervention - Post-intervention	mMRC dyspnea scale	↓ in I and C	Fidelity (interventionist): NR
				FVC	no changes	Acceptability: NR
				FEV1	no changes	Safety: NR
				DLCO	no changes	
				DLCO/VA	I ↑ C	
8	Manzetti, 1994 (United States)	LuTx candidates n = 21 22% male Age 40 (±10)		W_max_	no changes	Enrolment: 36/91 (40%) eligible for participation, 15/36 (42%) of eligible patients declined participation due to financial reasons (n = 10) or transport issues or inability to perform activities of daily living independently (n = 5)
		I: n = 5 % Male NR Age NR	I: 6-week, health education program + supervised aerobic training (treadmill, bicycle ergometer, 30 min/session, 2x/week, around aerobic threshold or 80% maximal ventilation) + strength training of upper extremity (low intensity)	6MWT	↑ in I and C, I ≈ C	Attrition: 9/21 (43%) completed; drop-outs due to Tx (n = 9) or hospitalization (n = 3). Number of drop-outs per group NR
		C: n = 4 % male NR Age NR	C: 6-week, health education program	QoL (QWB, QLI, SFSD)	↑ or ≈ I and C, I ≈ C	Fidelity (participants): NR
			Assessments: - Pre-intervention - Post-intervention			Fidelity (interventionist): NR
						Acceptability: NR
						Safety: NR

I, Intervention group; C, comparator group; Tx, transplantation; NR, not reported; 1RM, one-repetition maximum; 6MWD, six-minute walking distance; 6MWT, six-minute walking test; BiVAT, biventricular assist device; COPD, chronic obstructive pulmonary disease; DLCO/VA, alveolar volume ratio of carbon-monoxide diffusion capacity; DLCO, diffusion capacity of the lung for carbon monoxide; EMG, electromyography; FEV1, forced expiratory volume in 1 second; HRmax, maximal heart rate; HTx, heart transplantation; IMT, inspiratory muscle training; IVC, inspiratory vital capacity; LiTx, liver transplantation; LuTx, lung transplantation; LVAT, left ventricular assist device; MEP, maximal expiratory pressure; MIP, maximal inspiratory pressure; MLwHFQ, Minnesota Living with Heart Failure Questionnaire; mMRC dyspnea scale, modified Medical Research Council dyspnea scale; NA, Not applicable; NR, not reported; NT-proBNP, N-terminal prohormone of brain natriuretic peptide; P_a_CO_2_, partial pressure arterial carbon dioxide; P_a_O_2_, partial pressure arterial oxygen; PImax, maximal inspiratory pressure; QOL, quality of life; RPE, rate of perceived exertion; SpO_2_, saturation of peripheral oxygen; T_C_PCO_2_, transcutaneously measured pressure of arterial carbon dioxide; VO_2_peak, peak or maximal oxygen consumption; W_max_, peak work rate at the end of a cardiopulmonary exercise test; QWB, Quality of Well-being scale; QLI, Quality of Life Index; SFSD, Symptom Frequency/Symptom distress scale.

**TABLE 3:** Summary of RCTs- Nutritional interventions.

	First author, year (country of origin)	Sample characteristics Tx-type, total N, n per group, % male, age (y) (mean (sd) or median (range))	Intervention(s) and measurement points	Effectiveness Outcomes	Results – Effectiveness outcomes ↑ = significant increase ↓ = significant decrease ≈ no difference	Results – feasibility outcomes
1	Grat, 2017 (Poland)	Liver Tx candidates N = 55		90-day mortality rate	I ≈ C	Enrolment: 209/491 (43%) eligible for participation; 55/209 (26%) of eligible patients participated. Refusal to participate probably due to administrational factors
		I n = 26 81% male Age 52 (47–58)	I: once daily intake of a 4-strain probiotic preparation before breakfast (ProBacti 4 Enteric®: 3 × 10^9^ colony-forming units of *Lactococcus lactis* PB411 (50.0%), *Lactobacillus casei* PB121 (25.0%), *Lactobacillus acidophilus* PB111 (12.5%), and *Bifidobacterium bifidum* PB211 (12.5%) from enrolment until transplantation. Duration of intervention was <2–>10 weeks depending upon timing Tx	30-day and 90-day infection rate	I ↓ C	Attrition: 50/55 ((91%) completed (I 24/26 (92%); C 26/29 (90%)). Dropouts (n = 5) all discontinued treatment Post-Tx outcomes available of I: 21/26 (81%) and C: 23/29 (79%)
		C n = 29 74% male Age 50 (35–61)	C: placebo	5-days post-Tx: - AST - ALT - Bilirubin concentration - INR	I ↑ C I ↑ C I ↓ C I ≈ C	Fidelity (participants): I 2/26 (8%) and C: 3/29 (10%) discontinued treatment
			Assessments: - Baseline - Pre-Tx: follow-up with intervals of 10 weeks - Post-Tx: 90 days follow-up	Pre-transplant: - Waitlist mortality - Hospitalizations - Infections - Complications	None I ≈ C I ≈ C I ≈ C	Fidelity (interventionist): NR
				Post-transplant - Primary non-function - Early allograft dysfunction - Complications	I ≈ C I ≈ C I ≈ C	Acceptability: NR
				MELD-score changes	I ≈ C	Safety: NR
				CTP changes	I ≈ C	
2	Plank, 2015 (New Zealand)	Liver Tx candidates N = 101		Body composition - Body weight (kg) - Total body protein - Total body fat	I ≈ C I ≈ C I ≈ C	Enrolment: NR
		I n = 52 Male 63% Age 53 (25–68)	I: daily intake of immuno-nutrition, two 74 g sachets per day until the day of transplant, consisting of 7.5 g arginine, 2 g omega-3 fatty acids + 0,8g Ribonucleic acid. for 56–65 days (median)	Muscle function - Hand grip strength - Respiratory muscle strength	I ≈ C I ≈ C	Attrition: 101/120 (84%) completed (I 52/60 (87%); C 49/60 (82%)). Dropouts: I: delisting (n = 8), C: death (n = 4), delisting (n = 7)
		C n = 49 Male 73% Age 50 (22–59)	C: daily intake with a similar amount of an isocaloric, but not isonitrogenous, control product	Plasma phosphatidyl-choline fatty acids	I ↑ C at pre-Tx and day 10 measurements	Fidelity (participants): NR
			Assessments: - Baseline - Prior to Tx - 10, 30, 90, 180, 360 days after Tx	Fatigue (NR)	I ≈ C	Fidelity (interventionist): NR
				Graft rejection	I ≈ C	Acceptability: NR
				Length of stay at ICU	I ≈ C	Safety: intolerance to immune-nutrition in four participants
				Length of stay at hospital	I ≈ C	
3	Eguchi, 2011 (Japan)	Living donor Liver TX candidates N = 50		Infectious complications	I ↓ C	Enrolment: NR
		I n = 25 52% male Age 56 (33–66)	I group 1: 2 days preoperative and group 2: 2 weeks post-operative synbiotic therapy (*Bifidobacteriu breve*, *Lactobacillus casei* and *Galactooligosa charides* **)**	Mortality	I ≈ C	Attrition: NR
		C n = 25 64% male Age 57 (25–68)	C: placebo	Length of stay at ICU	I ≈ C	Fidelity (participants): NR
			Assessments: Not specified	Length of stay at hospital	I ≈ C	Fidelity (interventionist): NR
						Acceptability: NR
						Safety: all participants tolerated synbiotic therapy
4	Park, 2003 (United States)	Heart Tx candidates with BMI > 25 kg/m^2^ N = 43	All participants had one consultation session by a graduate student in clinical psychology under the supervision of the study’s registered dietitian, who provided the recommendations such as energy balance	Body weight change	I ↑ C	Enrolment: 43/54 (80%) of referred patients
		I n = 21 81% male Age 47.8 (±8.5)	I : 3-months weight-loss program comprised of bibliotherapy (written, 20-page manual containing brief lessons about cognitive and behavioral weight loss strategies), and telephone-based counseling (1x/week, 15–20 min) delivered by a therapist who has a bachelor’s or master’s degree in psychology.			Attrition: 36/43 (84%) completed (I 17/21 (81%); C 19/22 (86%)
		C n = 22 68% male Age 48.1 (±9.4)	C: 3-months weight-loss program comprised of bibliotherapy without counseling			Fidelity (participants): I returned more food diaries than C, but not significant; I returned more postcards than C, but not significant
			Assessments: - Pre-intervention - Post-intervention			Fidelity (interventionist): NR
						Acceptability: NR
						Safety: NR
5	Forli, 2001(a) (Norway)	Lung Tx candidates N = 65		Change in body weight.	I ↑ C1 and C2	Enrolment: 6/71(8%) of eligible patients excluded for various reasons: refused intervention (n = 1), dietary wishes (n = 1), absent during night/weekend (n = 1), short hospital stay (n = 1), death (n = 1).
		I n = 18 44% male Age 49 (44–53)	I: intensified nutritional support comprised of energy-rich diet and supplements, provided by a dietician during hospital stay	BMI (kg/m^2^)	C1 ↓ I and C2	Attrition: 49/65 (75%) completed. Drop-outs due to: not willing to record data (n = 1), missing data (n = 3), oedema (n = 2), death (n = 1)
		C1 n = 19 53% male Age 48 (44–52)	C1: normal hospital diet	Total energy intake/kg	C2 ↓ I and C1	Fidelity (participants): NR
		C2 n = 28 43% male Age 51 (48–55)	C2: normal weight lung Tx candidates	Total energy intake/REE predicted	I ↑ C1 and C2, C1 ↑ C2	Fidelity (interventionist): NR
			Assessments: - During hospitalization for lung Tx screening, exact moments NR			Acceptability: NR
						Safety: NR
6	Forli, 2001(b) (Norway)	Lung Tx candidates with underweight N = 71		Body composition - Change in body weight - Change in Fat mass - Change in Fat free mass	↑ in I (+2.9 kg) and C1 (+2.3 kg) group, not in C2 group I ↑ C1 ≈ C2 C1 ↑I ≈ C2	Enrolment: NR
		I n = 21 48% male Age 47 (28–59)	I: intensified sessions dietary counselling with suggestions for individual meal plans facilitating weight gain, booklet with dietary information and recipes, supplements, and support by telephone by the dietitian each month after hospital discharge. Mean intervention time was 22 weeks.	Blood samples - Albumin concentration - Phosphate concentration	I ≈ C1 ≈ C2 I ≈ C1 ≈ C2	Attrition: 54/71 (76%) completed (1 18/21 (86%); C1 13/21 (62%); C2 23/29 (79%)). Dropouts: I and C1 death (n = 8), Tx (n = 3), infection (n = 14); C2 death (n = 2), Tx (n = 4), infection (n = 7)
		C1 n = 21 48% male Age 46 (25–60)	C1: one session of individual dietary counselling with the dietitian. No follow-ups. The mean intervention time was 20 weeks.	Lung function test: - PaO_2_ - PaCO_2_ - FVC - FEV1 - TLCO	I ↓ C1 ≈ C2 I ≈ C1 ≈ C2 I ≈ C1 ≈ C2 I ≈ C1 ≈ C2 I ≈ C1 ≈ C2	Fidelity (participants): NR
		C2 n = 29 41% male Age 52 (26–60)	C2: normal weight Lung Tx candidates	Exercise testing: - handgrip strength - 6MWT	I ≈ C1 ≈ C2 I ≈ C1 ≈ C2	Fidelity (interventionist): NR
			Assessments: - Pre-intervention - 4–5 months after discharge			Acceptability: NR
						Safety: NR
7	Le Cornu, 2000 (United Kingdom)	Liver Tx candidates N = 82	No information who provided the advice in both groups nor the number of sessions	Biochemical parameters - Bilirubin - Creatinine - Urea - Alkaline phosphatase - Aspartate transaminase - INR	≈ within ≈ within I and C ↑ within I; ≈ within C ≈ within I; ↓ within C ≈ within I and C ≈ within I; ↑ within C	Enrolment: 116/328 (35%) patients were eligible, 82/116 (71%) of eligible patients consented
		I n = 42 69% male Age 52 (27–67)	I: Standard dietary advice to increase energy intake on top of the dietary recommendations they already had to follow for underlying medical conditions and daily enteral supplementation (750 calories out of 20 g protein and 33.5 g fat).	Anthropometric measurements: - Mid-arm circumference - Triceps skinfold thickness	I ≈ C I ≈ C	Attrition: 80-28 (98%) completed (I 41/42 (98%); C 39/40 (98%)). Dropouts due to I: lost to follow-up n = 1); C: delisted (n = 1)
		C n = 40 79% male Age 50 (24–68)	C: Standard dietary advice to increase energy intake on top of the dietary recommendations they already had to follow for underlying medical conditions.	Handgrip strength	I ≈ C	Fidelity (participants): NR
			Assessments: - Screening - Monthly follow-up until Tx or death	Energy Intake	I ≈ C	Fidelity (interventionist): NR
				Survival (pre-transplant)	I ≈ C	Acceptability: NR
				Days on ventilatory support	I ≈ C	Safety: NR
				Length of ICU stay	I ≈ C	
				Length of Hospital stay	I ≈ C	

I, Intervention group; C, comparator group; Tx, transplantation; NR, not reported; AST, asparate; ALT, alanine aminotransferase; INR, Internationalized Normalized Ratio; MELD, Model for End-stage Liver Disease; CTP, Chil-Turcotte-Pugh; ICU, Intensive Care Unit; BMI, Body Mass Index; PaO_2_, Arterial O_2_; PaCO_2_, Arterial CO_2_; FVC, Forced Vital Capacity; FEV1, Forced Expiratory Volume/1s; TLCO, Lung transfer factor carbon monoxide; 6MWT, six Minutes Walking Test.

**TABLE 4:** Summary of RCTs - Psychosocial interventions.

	First author, year (country of origin)	Sample characteristics Tx-type, total N, n per group, % male, age (y) (mean (sd) or median (range))	Intervention(s) and measurement points	Effectiveness Outcomes	Results – Effectiveness outcomes ↑ = significant increase ↓ = significant decrease ≈ no difference	Results – feasibility outcomes
1	Napolitano, 2002 (United States)	Lung Tx candidates N = 71		Health-related quality of life (SF36, PQLS)	SF36: I ↑ C on overall quality of life, mental health, role limitations due to emotional functioning, and vitality score PQLS: I ↑ C on overall score and subscales psychological functioning and physical functioning	Enrolment: 81/91 (89%) of eligible patients
		I n = 36 31% male Age 44.2 (±12.7)	I: 8-weeks, weekly, telephone-based psychological treatment comprised of supportive counselling and CBT, delivered by clinical psychology graduate	Anxiety (GHQ) Depression (GHQ)	I ↓ C on total score and subscales scores (anxiety, depression, social dysfunction, and somatic symptoms)	Attrition: 71/81 (88%) completed baseline (n = 2 delisted, n = 1 Tx, n = 6 withdrew consent, n = 1 died). 66/71 (93%) completed follow-up (I 34/36 (94%), missing data due to Tx (n = 2); C 32/35 (91%), missing data due to loss to follow-up (n = 3))
		C n = 35 31% male Age 46.6 (±12.4)	C: care as usual	Social support (PSSTx)	I ↑ C	Fidelity (participants): all participants received all sessions
			Assessments: - Pre-intervention - Post-intervention	Distress (PSTx)	I ≈ C	Fidelity (interventionist): NR
						Acceptability: NR
						Safety: NR
2	Rodrigue, 2005 (United States)	Lung Tx candidates N = 35		Quality of Life (QOLI)	I ↑ C at 1 and 3-month follow-up	Enrolment: 35/58 (60%) of eligible patients
		I n = 17 35% male Age 48.8 (±10.0)	I: 8–12-weeks, weekly, telephone-based CBT delivered by clinical psychology graduate students and interns	Mood (POMS)	I ↓ C at 3 month follow-up	Attrition: 35/35 (100%) at baseline, 31/35 (89%) completed all assessments
		C n = 18 33% male Age 49.0 (±11.3)	C: supportive treatment, delivery NR	Social intimacy (MSIS)	I ↑ C at 1 month follow-up	Fidelity (participants): I: 88% full treatment, C: 89% full treatment
			Assessments: - Pre-intervention - 1 month post-intervention - 3 month post-intervention	FEV1	I ≈ C	Fidelity (interventionist): NR
				Physical functioning (6MWT)	I ≈ C	Acceptability: I: high levels of comfort, rapport, helpfulness and convenience, low levels of distraction. 87% would participate again, 32% preference telephone counselling
						Safety: NR
3	Sharif, 2005 (Iran)	Liver Tx candidates N = 110		Health-related Quality of life (CLDQoL)	I ↑ scores on domains fatigue, emotional function, and total QOL score at 1-month follow-up. I ↑ on all domains of QoL at 3-month follow-up	Enrolment: NR
		I n = 55 76% male Age NR	I: 4-weeks, three individual sessions and one group session, 90 min/week, psycho-educational treatment, mode of delivery NR		Comparison with control group not reported	Attrition: NR
		C n = 55 75% male Age NR	C: educational booklet			Fidelity (participants): NR
			Assessments: - Pre-intervention - Post-intervention			Fidelity (interventionist): NR
						Acceptability: NR
						Safety: NR
4	Blumenthal, 2006 (United States)	Lung Tx candidates N = 328		Health-related Quality of life (PQLS, SF36, GHQ)	PQLS: I ≈ C SF36: I ↑ C on PSC and subscales mental health and vitality; I ≈ C on subscales general health, physical functioning, pain, physical role GHQ: I ↑ C	Enrolment: 389/625 (62%) of eligible patients. Drop out after randomization I: 34/200 (17%); C 27/189 (14%) due to death, Tx or delisting
		I n = 166 45% male Age 50 (±11)	I: 12-weeks, telephone-based, 30 min/week, supportive counseling and training in cognitive-behavioral coping skills coping skills delivered by trained social worker or psychologists	Anxiety (STAI)	I ↓ C	Attrition: I: 126/166 (76%) and C: 147/162 (91%) completed all assessments
		C n = 162 43% male Age 50 (±12)	C: care as usual	Depression (BDI)	I ↓ C	Fidelity (participants): 10.6 out of 12 sessions, 77% completed all sessions
			Assessments: - Pre-intervention - Post-intervention	Perceived stress (PSS)	I ≈ C	Fidelity (interventionist): 97.6% adhered to the protocol
				Life-orientation (LOT-R)	I ↑ C	Acceptability: NR
				Social Support (PSSC)	I ≈ C	Safety: no adverse events
				Shortness of breath (SDS-BQ)	I ≈ C	
				Survival pre-transplant	I ≈ C	
5	Rodrigue, 2011 (United States)	Kidney Tx candidates N = 62		Health-related Quality of life (QoLI, SF-36)	QoLI: I1 ↑ (clinical relevant) C, I2 ≈ C SF36: I1 ↑ (clinical relevant) C, I2 ≈ C	Enrolment: 65/110 (59%) of eligible patients (n = 18 excluded, n = 27 refused)
		I1 n = 22 64% male Age 53.2 (±11.1)	I1: 8-weeks,in person, 50 min/week, QoL-therapy, delivered by trained social workers or psychologists	Mood (POMS)	I1 ↑ (clinical relevant) C, I2 ≈ C	Attrition: 62/65 (95%) completed baseline, 51/62 (82%) completed all assessments
		I2 n = 20 40% male Age 48.6 (±11.9)	I2: 8-weeks, in person, 50 min/week, supportive care, delivered by trained social workers or psychologists	Distress (HSCL)	I1+ I2	Fidelity (participants): I1 17/22 (77%) and I2 17/20 (85%) received full treatment
		C n = 20 45% male Age 52.7 (±12.7)	C: care as usual	Social Intimacy (MSIS)	I1 ↑ (clinical relevant) C, I2 ≈ C	Fidelity (interventionist): NR
			Assessments: - Pre-intervention - 1-week post-intervention - 12-week post-intervention	No mental unhealthy days	I1 ≈ I2 ≈ C	Acceptability: high level of comfort, rapport, supportiveness and overall helpfulness
						Safety: NR
6	Gross, 2017 (United States)	Kidney and kidney pancreas Tx candidates N = 63		Health-related quality of life (SF36)	I ↑ C on MCS at 6-month follow-up I ↑ within group PCS score at 2-month follow-up	Enrolment: 63/388 (16%) of eligible patients
		I n = 32 43% male Age 50 (±12)	I: 8-weeks, group-based, combined in-person and telephone-based, mindfulness stress reduction training, delivered by a certified mindfulness-based stress reduction teacher	Anxiety (STAI)	I ≈ C	Attrition: 51/63 (81%) completed assessment at 2 months after baseline (I 27/32 (84%); C 24/31 (77%)). 42/63 (67%) completed all assessments (I 22/32 (69%); C 20/31(65%))
		C n = 31 43% male Age 50 (±12)	C: 8-week, group based, combined in-person and telephone-based, weekly, structured support group, delivered by a group facilitator	Depression (CES-D)	I ↑ C at 2-month follow-up I ≈ C at 6-month follow-up	Fidelity (participants): attendance seven out of eight sessions; in both groups; n = 4 never attended
			Assessments: - Baseline - 2 months after baseline - 6 months after baseline	Sleep quality (PSQI)	I ≈ C	Fidelity (interventionist): no treatment contamination found
				Pain (SF12 pain item)	I ≈ C	Acceptability: 90% reported continuing meditation practices, 67%–80% indicated that MBSR was helpful
				Fatigue (PROMIS-Fatigue SF)	I ≈ C	Safety: No intervention-related adverse events occurred

Tx, transplant; I, intervention group; C, comparator group; NR, Not reported; CBT, cognitive behavioural therapy; SF-36, short form 36 questionnaire; GHQ, general health questionnaire; PQLS, pulmonary-specific quality-of-life-scale; PSSTx, perceived social support related to transplantation; PSTx, perceived stress related to transplantation QOLI, quality of life inventory; POMS, Profile of Mood States Short-Form; MSIS, 17-item Miller Social Intimacy Scale; FEV1, Forced expiratory volume; 6MWT, six minute walk test; CLDQoL, Chronic Liver Disease Quality of Life; PQLS, pulmonary specific Quality of Life Scale; STAI, State-Trait Anxiety Inventory- State form; BDI, Beck Depression Inventory; PSS, perceived stress scale; LOT-R, life orientation test- revised; PSSC, Perceived social support scale; SDS-BQ, University of California San Diego Shortness of breath Questionnaire; COPE Inventory; POMS, Profile of Mood States-Short Form; HSCL, Hopkins Symptom Checklist-25; No of unhealthy mental health days in the past month, subjective reporting of number of days experiencing stress; depression or anxiety in the past month; CES-D, The Centre for Epidemiologic Studies Depression Scale; PSQI, The Pittsburg Sleep Quality Index; SF-12, Short-Form 12 questionnaire; MCS, Mental Composite Score of the SF-12; PCS, Physical Composite Score of the SF-12; PROMIS-Fatigue, PROMIS-Fatigue Short Form v1.

## Results

A total of 4 PICO questions were identified, along with key criteria for analysis, as presented in Table 1. The systematic review of literature yielded 34 studies on exercise, 7 on nutritional, and 10 on psychological interventions (Figures 1–3). Summaries of the literature evidence were generated and are presented in Tables 2–4 and Supplementary File S1. A total of 26 recommendations were formulated (Supplementary File S2). At the Consensus Conference, the literature summaries and recommendations were presented, discussed, and amended according to the ESOT consensus-finding process. In response to the considerations voiced during the discussion, and in an attempt to avoid overlap, the number and nature of the recommendations was revised to 10 well-defined recommendations, i.e., 4 general and 6 specific ones. In a first voting round, 100% agreement was achieved on 7 out of 10 recommendations, whereas 3 recommendations reached 86% agreement (1.2, 2.4, 2.5) due to being considered as too exclusive of certain patient groups. Consensus was reached to amend these recommendations to be more inclusive, and in a second voting round, 100% agreement was achieved on all 10 recommendations.

## Recommendations

### PICO Question 1

In adult candidates for lung, liver, kidney, & heart transplantation: what is the evidence for the effectiveness of pre-transplant exercise training, nutritional support and psychosocial interventions as measured by prehabilitation efficacy outcomes, clinical outcomes and patient-reported outcomes.

To date, multimodal prehabilitation programs that offer a combination of exercise, nutritional, and psychosocial interventions, have not been studied in solid organ transplant candidates. Rather, literature is limited to studies investigating a single type of intervention. Based on the committee’s literature review and analysis of the predefined criteria prehabilitation effectiveness, clinical and patient-reported outcomes, one general recommendation and two specific recommendations were made.

#### Recommendation 1.1

Studies are needed that evaluate multi-modal prehabilitation interventions in candidates for all types of solid organ transplantation and that focus on core outcomes and implementation. Such studies should be of high quality, and preferably–but not exclusively–adequately powered RCTs.

Quality of Evidence: not applicable.

Strength of Recommendation: Strong.

Rationale: Although supportive, the current evidence (Table 2–4) based on the effectiveness of pre-transplant exercise, nutritional, and psychosocial interventions is weak because of the limited number of randomized studies; 8 for exercise interventions [19–25], 7 for nutritional interventions [26–32], and 6 for psychosocial interventions [33–38]. In addition, 25 non-randomized studies regarding exercise [39–64], and 4 non-randomized studies on psychosocial interventions [65–68] were retrieved by literature review (Supplementary File S1). The small sample size per study and the limited size and heterogeneity of the total populations studied, the variability in interventions and outcomes measures, the generally low-to-moderate quality of the methodology, and–as a result–the inconsistency of findings across studies (Tables 2–4), warrants high-quality studies on multimodal prehabilitation before solid organ transplantation.

#### Recommendation 1.2

It is suggested that exercise-based interventions are included in the prehabilitation care of solid organ transplant candidates, with the objective to improve cardiorespiratory fitness and/or inspiratory muscle strength.

Quality of Evidence: Low.

Strength of Recommendation: Weak.

Rationale: Although the number and size of RCTs is limited, studies have shown that exercise training was associated with clinically meaningful improvement in cardiorespiratory fitness in heart transplant candidates [19, 21, 22, 24] and a clinically meaningful gain in inspiratory muscle strength in heart and in liver transplant candidates [19, 23, 24].

#### Recommendation 1.3

It is suggested that probiotic therapy be used in candidates for liver transplantation to reduce their susceptibility to post-transplant infections.

Quality of Evidence: Very low.

Strength of Recommendation: Weak.

Rationale: Two studies were identified in which pre-transplant probiotic and symbiotic therapy were associated with reduced post-transplant infection rates in recipients of a liver transplant [26, 28]. However, both studies had small sample sizes (*n* = 44/*n* = 50) and used different products.

### PICO Question 2

In adult candidates for lung, liver, kidney, & heart transplantation: which type(s) of exercise, nutritional support and psychosocial interventions are recommended in the pre-transplant phase?

As there are no established prehabilitation programs for solid organ transplant candidates, evidence review was focused on studies that addressed interventions that could be of value in a multimodal prehabilitation program. One general recommendation and four specific recommendations were established.

#### Recommendation 2.1

Studies are needed to identify the optimal component(s) and the mode of delivery of pre-transplant multimodal prehabilitation programs in solid organ transplant candidates. Such studies should be of high quality and be preferably -but not exclusively-adequately powered RCTs.

Quality of Evidence: not applicable.

Strength of Recommendation: Strong.

Rationale: Because of the heterogeneity in the study populations and in the nature and delivery mode of the interventions described in the current literature (Tables 2–4; Supplementary File S1), it remains unclear which organ transplant candidates would benefit most from which intervention program. Most exercise intervention studies used aerobic training [19–22, 24], peripheral muscle training [41], inspiratory muscle strength training [25, 51], or a combination of these training modalities. Nutrition intervention studies mostly used nutritional support to optimize energy intake and/or obtain weight loss [29–32, 69, 70]. Whilst psychosocial interventions predominantly included cognitive behavioral therapy [33, 34, 36, 37, 65, 67], psycho-educational interventions [35, 68] and stress management and relaxation techniques [38, 67] or a combination of these interventions. Studies are needed that will help determine the modalities of the intervention, and for each modality (exercise, nutrition or psychosocial), the intervention characteristics (frequency, intensity and timing), the delivery mode (type of interventionist, level of supervision, home-based versus in- or outpatient) for each type of donor organ recipient.

#### Recommendation 2.2

Solid organ transplant candidates who are underweight may be offered nutritional interventions with the aim to achieve optimal target weight before the transplant.

Quality of Evidence: Very low.

Strength of Recommendation: Weak.

Rationale: Evidence from two intervention studies in lung transplant candidates [29, 30] have indicated that increased caloric intake before transplantation may allow solid organ transplant candidates, especially those who are underweight, to reach a pre-transplant target weight. However, these studies had a small sample size and were conducted in different settings (hospital vs. outpatient clinic).

#### Recommendation 2.3

Solid organ transplant candidates who are overweight may be offered nutritional interventions with the aim to achieve optimal target weight before the transplant.

Quality of Evidence: Very low.

Strength of Recommendation: Weak.

Rationale: One study (*n* = 43) [32] showed that a weight-loss program, consisting of bibliotherapy and voice call counselling by a dietician, was successful in reducing body weight in adult candidates for heart transplantation.

#### Recommendation 2.4

It is suggested that cognitive behavioral therapy and psychoeducational interventions are considered for solid organ transplant candidates who have symptoms of anxiety and/or depression.

Quality of Evidence: Very low.

Strength of Recommendation: Weak.

Rationale: Six studies utilized elements of cognitive behavioral therapy (CBT) and psychoeducational interventions [33, 34, 36, 37, 65, 67] of which five reported a significant decrease in symptoms of anxiety and depression or mood [33, 34, 36, 37, 65] in lung, liver, and kidney transplant candidates. However, studies differed regarding duration (8–12 weeks), modality (group vs. individual; remote vs. in person), and most studies had small sample sizes (*n* = 29 to *n* = 71) (Table 4). Only the study of Blumenthal et al (2006)) [36] had an adequate sample size (*n* = 328).

#### Recommendation 2.5

It is suggested to consider stress-reducing interventions such as mindfulness-based stress reduction or relaxation techniques in candidates for solid organ transplantation to reduce anxiety or stress levels.

Quality of Evidence: Very low.

Strength of Recommendation: Weak.

Rationale: In two studies among kidney and kidney-pancreas transplant candidates, stress-reducing interventions were associated with alleviated symptoms of anxiety [65] or depression [38, 65] directly after the intervention. However, this effect was not maintained long-term. In addition, sample sizes were small (*n* = 41/*n* = 63) and the intervention differed regarding content and interventionist.

### PICO Question 3

In adult candidates for lung, liver, kidney, & heart transplantation: what are the outcomes relevant to exercise and physical activity, nutritional support and psychosocial interventions that should be measured pre-transplant?

In order to reliably assess the effects of prehabilitation interventions, it is imperative to standardize outcome measures, their definitions, and the tools to measure them. Literature was reviewed with respect to the outcomes evaluated as well as the tools to measure them. One general recommendation was formulated.

#### Recommendation 3.1

It is strongly recommended that a *core outcome measurement set* is defined for future multimodal prehabilitation studies in solid organ transplant candidates.

Quality of Evidence: not applicable.

Strength of Recommendation: Strong.

Rationale: The studies retrieved during this review varied widely with respect to the clinical and patient-reported outcomes that were utilized, and the methods to assess them (Tables 2–4). Most exercise intervention studies included cardiorespiratory fitness including peak or maximal oxygen consumption (VO_2_peak) and/or six-minute walking distance (6MWD)], Health-related Quality of Life (HRQoL), dyspnea, or maximal inspiratory pressures outcome measures. Nutritional intervention studies mostly monitored weight changes, infection rates, body composition and survival as either primary or secondary outcomes. The outcomes in studies that used psychosocial interventions included mostly HRQoL as well as parameters of mood, social intimacy and coping, while the use of clinical outcomes was rare. All stakeholders including solid organ transplant candidates and recipients, transplant professionals, and researchers in the field of transplantation strongly supported that a *core outcome set* be defined to facilitate comparative studies and give impetus to the field. A *core outcomes set* refers to a minimum set of outcome measures that are critical to patients, caregivers, and health professionals for decision making [71]. Selected outcomes that have so far not been considered but do carry clinical relevance during the pre-transplant waiting time are health-related physical fitness parameters such as muscular fitness, motor fitness, body composition and (cardio)metabolic health, as well as patient-reported outcomes such as fatigue, medication adherence and lifestyle, and clinical outcomes such as duration of intensive care stay, hospitalization, (re-)admissions, complications, graft function and survival, and waitlist and post-transplant mortality.

### PICO Question 4

In adult candidates for lung, liver, kidney, & heart transplantation: what is the evidence for the feasibility (enrolment, retention, acceptability, fidelity, safety) of prehabilitation?

Implementation of prehabilitation in clinical practice of solid organ transplantation should be supported by evidence of feasibility. Two systematic reviews have previously concluded that exercise prehabilitation is feasible and safe for solid organ transplant candidates [11, 12]. The review by Wallen et al was performed with focus on the feasibility outcomes enrolment, retention, acceptability, fidelity and safety [11]. One general recommendation was made.

#### Recommendation 4.1

It is strongly recommended that future studies on multimodal prehabilitation in solid organ transplant candidates include the specific assessment of feasibility.

Quality of Evidence: Moderate.

Strength of Recommendation: Strong.

Rationale: One study was identified that was specifically designed to assess the feasibility of delivering a psychosocial prehabilitation in solid organ transplant candidates. This study showed that a stress management and relaxation training program in liver transplant candidates was efficiently deliverable and considered acceptable and tolerable by the patients [67]. However, the enrolment rate was low, (29%) and the attrition rate was moderate (68%). Amongst the remainder of the literature, most studies reported on some aspect(s) of feasibility as a secondary outcome, mainly regarding enrolment and attrition (Tables 2–4). The feasibility measures fidelity of participants and/or interventionist and safety were less reported (Tables 2–4). For the exercise intervention studies, the enrolment rate was approximately 86%, while the average attrition rate ranged between 71% and 100% [19–25]. However, drop-outs were often due to transplant surgery. In the studies on nutritional interventions, feasibility measures were poorly reported. If reported, the enrolment rate was found to be low to moderate [26, 31, 32]. Attrition rates ranged between 62% and 98% [26, 27, 29–32]. In the psychosocial intervention studies, enrolment rates ranged between 24% and 59%, attrition rates between 69% and 88%, and acceptability of the intervention was high [33, 34, 36–38]. Only two studies reported the occurrence of adverse events [27, 65], but no serious adverse events occurred.

Overall, the consensus was that these studies do support the notion that it is feasible, acceptable and safe for adults to participate in exercise, nutritional, and psychosocial interventions during the waiting-list period (Tables 2–4). Although enrolment in studies differed significantly across studies, the overall willingness to participate in studies was found to be good and the attrition rates are adequate, and few adverse events are reported. Fidelity of participants as well as the interventionist and acceptability of the intervention are less reported. Nonetheless, implementation of prehabilitation in a clinical practice has not been established so far. Future dedicated studies should focus on the feasibility of implementation in clinical practice by assessing factors related to potential implementation strategy effects (e.g., adoption, fidelity, reach, sustainability) and factors to inform the design or development of the implementation strategy (e.g., acceptability, adaptability, feasibility, compatibility, complexity, self-efficacy, context, costs) [72].

## Discussion and Future Perspectives

The newly established ESOT consensus platform has proven successful in supporting the development of evidence-based consensus recommendations for prehabilitation in candidates for solid organ transplantation. Ten recommendations were formulated for which full consensus was reached within two voting rounds. This indicated that a high degree of consensus existed amongst all stakeholders from the prehabilitation, rehabilitation and transplantation fields, including transplant recipients and their representatives, and that the recommendations are a balanced representation of current published evidence and expert opinion.

Published evidence on prehabilitation before solid organ transplantation was found to be limited and consisted of studies addressing unimodal prehabilitation interventions with heterogeneous design, methodology and relatively small sample sizes. Nevertheless, by consensus and expert opinion, the available evidence on effects of prehabilitation on physical functioning, nutritional status, and psychosocial wellbeing and the evidence on safety of prehabilitation interventions was felt sufficiently strong to recommend that multimodal, patient-tailored prehabilitation should be offered as standard of care to patients awaiting solid organ transplantation. Specific recommendations included exercise-based intervention as well as psychological and stress management support for all solid organ transplant candidates, nutritional intervention for those who are over- or underweight, and probiotic supplementation for candidates for liver transplantation.

Because of the shortage in clinical evidence, however, particularly strong recommendations were formulated regarding the urgent need for high quality, but not exclusively, randomized controlled trials and implementation research studies that address the feasibility and effectiveness of pre-transplant multimodal prehabilitation. Two RCTs on multimodal prehabilitation interventions in kidney transplant candidates are currently underway: the FRAIL-MAR-study (NCT04701398) [73] and the PreCareTx-study (NCT05489432).

In addition, it was strongly recommended that priority should be given to the definition and consistent use of a Core Outcome Set to be measured by all future trials modalities, timing, duration and delivery modes of an optimal prehabilitation program.

From the in-person public discussions during the Consensus ESOT Conference, additional constructive perspectives emerged. It was advocated that clinical guidelines should be broadly applicable to transplant candidates irrespective of organ type, while leaving room for organ-specific recommendations, such as probiotics for liver transplant candidates. It was noted that intoxication-related interventions are not included in the recommendations, as intoxication (i.e., tobacco smoking or alcohol abuse) is typically addressed prior to patients joining the waitlist. The suggestion was made to formulate recommendations regarding pre-transplant peer support, however, such was considered premature as no evidence base could be found in the literature review. Lastly, the consideration was made that the designing of future studies or the future revisiting of the new guidelines may benefit of being informed by the prehabilitation literature in the broader field of surgery. However, unlike elective surgery, the waiting time is often unpredictably long while physical and mental condition may deteriorate due to the underlying disease. Therefore, prehabilitation should be offered throughout the waiting period from the moment of listing until transplantation.

These new evidence-based recommendations on prehabilitation serve to support best clinical practice in solid organ transplantation and help identify priorities for future research, thus optimizing patient health and post-transplant clinical outcome. The final recommendations will be included in the ESOT guidelines for transplant management, and under the auspices of the ESOT consensus development platform, will undergo continuous review and updating as new evidence becomes available.

## Data Availability

The original contributions presented in the study are included in the article/Supplementary Material, further inquiries can be directed to the corresponding author.

## Abbreviations

AGREE, Appraisal of Guidelines for Research & Evaluation; CET, Centre for Evidence in Transplantation; ESOT, European Society of Organ Transplantation; GRADE, Grading of Recommendations, Assessment, Development, and Evaluations; PICO, Population, Intervention, Comparator and Outcome; PRISMA, Preferred Reporting Items for Systematic reviews and Meta-Analyses; RCT, Randomized Controlled Trial.

## References

[1] Davison SN Levin A Moss AH Jha V Brown EA Brennan F Executive Summary of the KDIGO Controversies Conference on Supportive Care in Chronic Kidney Disease: Developing a Roadmap to Improving Quality Care. Kidney Int (2015) 88(3):447–59. 10.1038/ki.2015.110 25923985

[2] Kilic A Acker MA Atluri P. Dealing With Surgical Left Ventricular Assist Device Complications. J Thorac Dis (2015) 7(12):2158–64. 10.3978/j.issn.2072-1439.2015.10.64 26793336 PMC4703654

[3] Rocker G. Harms of Overoxygenation in Patients With Exacerbation of Chronic Obstructive Pulmonary Disease. CMAJ (2017) 189(22):E762–E763. 10.1503/cmaj.170196 28584039 PMC5461124

[4] Minnella EM Carli F. Prehabilitation and Functional Recovery for Colorectal Cancer Patients. Ejso-eur J Surg Onc (2018) 44(7):919–26. 10.1016/j.ejso.2018.04.016 29754828

[5] Carli F Scheede-Bergdahl C. Prehabilitation to Enhance Perioperative Care. Anesthesiol Clin (2015) 33(1):17–33. 10.1016/j.anclin.2014.11.002 25701926

[6] Heger P Probst P Wiskemann J Steindorf K Diener MK Mihaljevic AL. A Systematic Review and Meta-Analysis of Physical Exercise Prehabilitation in Major Abdominal Surgery (PROSPERO 2017 CRD42017080366). J Gastrointest Surg (2020) 24(6):1375–85. 10.1007/s11605-019-04287-w 31228083

[7] Moyer R Ikert K Long K Marsh J. The Value of Preoperative Exercise and Education for Patients Undergoing Total Hip and Knee Arthroplasty: A Systematic Review and Meta-Analysis. JBJS Rev (2017) 5(12):e2. 10.2106/JBJS.RVW.17.00015 29232265

[8] Waterland JL McCourt O Edbrooke L Granger CL Ismail H Riedel B Efficacy of Prehabilitation Including Exercise on Postoperative Outcomes Following Abdominal Cancer Surgery: A Systematic Review and Meta-Analysis. Front Surg (2021) 8:628848. 10.3389/fsurg.2021.628848 33816546 PMC8017317

[9] Lambert JE Hayes LD Keegan TJ Subar DA Gaffney CJ. The Impact of Prehabilitation on Patient Outcomes in Hepatobiliary, Colorectal, and Upper Gastrointestinal Cancer Surgery: A PRISMA-Accordant Meta-Analysis. Ann Surg (2021) 274(1):70–7. 10.1097/SLA.0000000000004527 33201129

[10] van Wijk L Bongers BC Berkel AEM Buis CI Reudink M Liem MSL Improved Preoperative Aerobic Fitness Following a Home-Based Bimodal Prehabilitation Programme in High-Risk Patients Scheduled for Liver or Pancreatic Resection. Br J Surg (2022) 109(11):1036–9. 10.1093/bjs/znac230 35851601 PMC10364722

[11] Wallen MP Skinner TL Pavey TG Hall A Macdonald GA Coombes JS. Safety, Adherence and Efficacy of Exercise Training in Solid-Organ Transplant Candidates: A Systematic Review. Transplant Rev (2016) 30(4):218–26. 10.1016/j.trre.2016.07.004 27496067

[12] Pesce de Souza F Massierer D Anand Raje U Tansey CM Boruff J Janaudis-Ferreira T. Exercise Interventions in Solid Organ Transplant Candidates: A Systematic Review. Clin Transplant (2020) 34(9):e13900. 10.1111/ctr.13900 32391965

[13] Janaudis-Ferreira T Mathur S Deliva R Howes N Patterson C Rakel A Exercise for Solid Organ Transplant Candidates and Recipients: A Joint Position Statement of the Canadian Society of Transplantation and CAN-RESTORE. Transplantation (2019) 103(9):e220–e238. 10.1097/TP.0000000000002806 31461743

[14] Cillo U Weissenbacher A Pengel L Jochmans I Roppolo D Amarelli C ESOT Consensus Platform for Organ Transplantation: Setting the Stage for a Rigorous, Regularly Updated Development Process. Transpl Int (2022) 35:10915. 10.3389/ti.2022.10915 36406781 PMC9667481

[15] Davies KS. Formulating the Evidence Based Practice Question: A Review of the Frameworks. Evid Based Libr Inf Pract (2011) 6(2):75–80. 10.18438/b8ws5n

[16] Guyatt GH Oxman AD Vist GE Kunz R Falck-Ytter Y Alonso-Coello P GRADE: An Emerging Consensus on Rating Quality of Evidence and Strength of Recommendations. Br Med J (2008) 336(7650):924–6. 10.1136/bmj.39489.470347.AD 18436948 PMC2335261

[17] DMDIAf. investopedia.com/terms/d/delphi-method.asp (2023). Available From: https://www.investopedia.com/terms/d/delphi-method.asp (Accessed May 11th, 2023).

[18] Brouwers MC Kho ME Browman GP Burgers JS Cluzeau F Feder G The Global Rating Scale Complements the AGREE II in Advancing the Quality of Practice Guidelines. J Clin Epidemiol (2012) 65(5):526–34. 10.1016/j.jclinepi.2011.10.008 22189163

[19] Laoutaris ID Dritsas A Adamopoulos S Manginas A Gouziouta A Kallistratos MS Benefits of Physical Training on Exercise Capacity, Inspiratory Muscle Function, and Quality of Life in Patients With Ventricular Assist Devices Long-Term Postimplantation. Eur J Cardiovasc Prev Rehabil (2011) 18(1):33–40. 10.1097/HJR.0b013e32833c0320 20571404

[20] Gloeckl R Halle M Kenn K. Interval Versus Continuous Training in Lung Transplant Candidates: A Randomized Trial. J Heart Lung Transplant (2012) 31(9):934–41. 10.1016/j.healun.2012.06.004 22884381

[21] Hayes K Leet AS Bradley SJ Holland AE. Effects of Exercise Training on Exercise Capacity and Quality of Life in Patients With a Left Ventricular Assist Device: A Preliminary Randomized Controlled Trial. J Heart Lung Transplant (2012) 31(7):729–34. 10.1016/j.healun.2012.02.021 22425235

[22] Adamopoulos S Gouziouta A Mantzouratou P Laoutaris ID Dritsas A Cokkinos DV Thyroid Hormone Signalling Is Altered in Response to Physical Training in Patients With End-Stage Heart Failure and Mechanical Assist Devices: Potential Physiological Consequences? Interactive Cardiovasc Thorac Surg (2013) 17(4):664–8. 10.1093/icvts/ivt294 PMC3781806 23820669

[23] Limongi V Dos Santos DC da Silva AMO Ataide EC Mei MFT Udo EY Effects of a Respiratory Physiotherapeutic Program in Liver Transplantation Candidates. Transpl Proc (2014) 46(6):1775–7. 10.1016/j.transproceed.2014.05.044 25131034

[24] Forestieri P Guizilini S Peres M Bublitz C Bolzan DW Rocco IS A Cycle Ergometer Exercise Program Improves Exercise Capacity and Inspiratory Muscle Function in Hospitalized Patients Awaiting Heart Transplantation: A Pilot Study. Braz J Cardiovasc Surg (2016) 31(5):389–95. 10.5935/1678-9741.20160078 27982348 PMC5144561

[25] Pehlivan E Mutluay F Balci A Kilic L. The Effects of Inspiratory Muscle Training on Exercise Capacity, Dyspnea and Respiratory Functions in Lung Transplantation Candidates: A Randomized Controlled Trial. Clin Rehabil (2018) 32(10):1328–39. 10.1177/0269215518777560 29843525

[26] Grat M Wronka KM Lewandowski Z Grat K Krasnodebski M Stypulkowski J Effects of Continuous Use of Probiotics Before Liver Transplantation: A Randomized, Double-Blind, Placebo-Controlled Trial. Clin Nutr (2017) 36(6):1530–9. 10.1016/j.clnu.2017.04.021 28506447

[27] Plank LD Mathur S Gane EJ Peng SL Gillanders LK McIlroy K Perioperative Immunonutrition in Patients Undergoing Liver Transplantation: A Randomized Double-Blind Trial. Hepatology (2015) 61(2):639–47. 10.1002/hep.27433 25212278

[28] Eguchi S Takatsuki M Hidaka M Soyama A Ichikawa T Kanematsu T. Perioperative Synbiotic Treatment to Prevent Infectious Complications in Patients After Elective Living Donor Liver Transplantation: A Prospective Randomized Study. Am J Surg (2011) 201(4):498–502. 10.1016/j.amjsurg.2010.02.013 20619394

[29] Forli L Pedersen JI Bjortuft O Vatn M Boe J. Dietary Support to Underweight Patients With End-Stage Pulmonary Disease Assessed for Lung Transplantation. Respiration (2001) 68(1):51–7. 10.1159/000050463 11223731

[30] Forli L Bjortuft O Vatn M Kofstad J Boe J. A Study of Intensified Dietary Support in Underweight Candidates for Lung Transplantation. Ann Nutr Metab (2001) 45(4):159–68. 10.1159/000046724 11463999

[31] Le Cornu KA McKiernan FJ Kapadia SA Neuberger JM. A Prospective Randomized Study of Preoperative Nutritional Supplementation in Patients Awaiting Elective Orthotopic Liver Transplantation. Transplantation (2000) 69(7):1364–9. 10.1097/00007890-200004150-00026 10798755

[32] Park TL Perri MG Rodrigue JR. Minimal Intervention Programs for Weight Loss in Heart Transplant Candidates: A Preliminary Examination. Prog Transpl (2003) 13(4):284–8. 10.1177/152692480301300408 14765721

[33] Napolitano MA Babyak MA Palmer S Tapson V Davis RD Blumenthal JA Effects of a Telephone-Based Psychosocial Intervention for Patients Awaiting Lung Transplantation. Chest (2002) 122(4):1176–84. 10.1378/chest.122.4.1176 12377839

[34] Rodrigue JR Baz MA Widows MR Ehlers SL. A Randomized Evaluation of Quality-Of-Life Therapy With Patients Awaiting Lung Transplantation. Am J Transplant (2005) 5(10):2425–32. 10.1111/j.1600-6143.2005.01038.x 16162191

[35] Sharif F Mohebbi S Tabatabaee HR Saberi-Firoozi M Gholamzadeh S. Effects of Psycho-Educational Intervention on Health-Related Quality of Life (QOL) of Patients With Chronic Liver Disease Referring to Shiraz University of Medical Sciences. Health Qual Life Outcomes (2005) 3:81. 10.1186/1477-7525-3-81 16356186 PMC1325272

[36] Blumenthal JA Babyak MA Keefe FJ Davis RD Lacaille RA Carney RM Telephone-Based Coping Skills Training for Patients Awaiting Lung Transplantation. J Consult Clin Psychol (2006) 74(3):535–44. 10.1037/0022-006X.74.3.535 16822110

[37] Rodrigue JR Mandelbrot DA Pavlakis M. A Psychological Intervention to Improve Quality of Life and Reduce Psychological Distress in Adults Awaiting Kidney Transplantation. Nephrol Dial Transplant (2011) 26(2):709–15. 10.1093/ndt/gfq382 20603243 PMC3108357

[38] Gross CR Reilly-Spong M Park T Zhao R Gurvich OV Ibrahim HN. Telephone-Adapted Mindfulness-Based Stress Reduction (tMBSR) for Patients Awaiting Kidney Transplantation. Contemp Clin Trials (2017) 57:37–43. 10.1016/j.cct.2017.03.014 28342990 PMC5512599

[39] Ben-Gal T Pinchas A Zafrir N Sahar G Berman M Aravot D. Long-Term Physical Training in Cardiac Transplant Candidates: Is it Feasible? Transpl Proc (2000) 32(4):740–2. 10.1016/s0041-1345(00)00964-7 10856566

[40] Karapolat H Engin C Eroglu M Yagdi T Zoghi M Nalbantgil S Efficacy of the Cardiac Rehabilitation Program in Patients With End-Stage Heart Failure, Heart Transplant Patients, and Left Ventricular Assist Device Recipients. Transpl Proc (2013) 45(9):3381–5. 10.1016/j.transproceed.2013.06.009 24182820

[41] Florian J Rubin A Mattiello R Fontoura FF Camargo Jde J Teixeira PJ. Impact of Pulmonary Rehabilitation on Quality of Life and Functional Capacity in Patients on Waiting Lists for Lung Transplantation. J Bras Pneumol (2013) 39(3):349–56. 10.1590/S1806-37132013000300012 23857680 PMC4075851

[42] Li M Mathur S Chowdhury NA Helm D Singer LG. Pulmonary Rehabilitation in Lung Transplant Candidates. J Heart Lung Transpl (2013) 32(6):626–32. 10.1016/j.healun.2013.04.002 23701852

[43] Debette-Gratien M Tabouret T Antonini MT Dalmay F Carrier P Legros R Personalized Adapted Physical Activity Before Liver Transplantation: Acceptability and Results. Transplantation (2015) 99(1):145–50. 10.1097/TP.0000000000000245 25531893

[44] Kenn K Gloeckl R Soennichsen A Sczepanski B Winterkamp S Boensch M Predictors of Success for Pulmonary Rehabilitation in Patients Awaiting Lung Transplantation. Transplantation (2015) 99(5):1072–7. 10.1097/TP.0000000000000472 25393161

[45] Pehlivan E Balci A Kilic L Kadakal F. Preoperative Pulmonary Rehabilitation for Lung Transplant: Effects on Pulmonary Function, Exercise Capacity, and Quality of Life; First Results in Turkey. Exp Clin Transplant (2018) 16(4):455–60. 10.6002/ect.2017.0042 28969527

[46] da Fontoura FF Berton DC Watte G Florian J Schio SM Camargo JDP Pulmonary Rehabilitation in Patients With Advanced Idiopathic Pulmonary Fibrosis Referred for Lung Transplantation. J Cardiopulmonary Rehabil Prev (2018) 38(2):131–4. 10.1097/HCR.0000000000000315 29465499

[47] Ochman M Maruszewski M Latos M Jastrzebski D Wojarski J Karolak W Nordic Walking in Pulmonary Rehabilitation of Patients Referred for Lung Transplantation. Transpl Proc (2018) 50(7):2059–63. 10.1016/j.transproceed.2018.02.106 30177109

[48] Byrd R Smith P Mohamedaly O Snyder LD Pastva AM. A 1-Month Physical Therapy-Based Outpatient Program for Adults Awaiting Lung Transplantation: A Retrospective Analysis of Exercise Capacity, Symptoms, and Quality of Life. Cardiopulmonary Phys Ther J (2019) 30(2):61–9. 10.1097/CPT.0000000000000087 30983916 PMC6456901

[49] Florian J Watte G Teixeira PJZ Altmayer S Schio SM Sanchez LB Pulmonary Rehabilitation Improves Survival in Patients With Idiopathic Pulmonary Fibrosis Undergoing Lung Transplantation. Sci Rep (2019) 9:9347. 10.1038/s41598-019-45828-2 31249363 PMC6597536

[50] McAdams-DeMarco MA Ying H Van Pilsum Rasmussen S Schrack J Haugen CE Chu NM Prehabilitation Prior to Kidney Transplantation: Results From a Pilot Study. Clin Transplant (2019) 33(1):e13450. 10.1111/ctr.13450 30462375 PMC6342659

[51] Kilic L Pehlivan E Balci A Bakan ND. Effect of 8-week Pulmonary Rehabilitation Program on Dyspnea and Functional Capacity of Patients on Waiting List for Lung Transplantation. Turkish Thorac J (2020) 21(2):110–5. 10.5152/TurkThoracJ.2019.18202 PMC7089708 32203001

[52] Pehlivan E Balci A Kilic L. The Effect of Pulmonary Rehabilitation on Dyspnea and Factors Related to Dyspnea in Lung Transplantation Candidates. Eur Res J (2020) 6(5):395–400. 10.18621/eurj.531507

[53] Lorenz EC Hickson LJ Weatherly RM Thompson KL Walker HA Rasmussen JM Protocolized Exercise Improves Frailty Parameters and Lower Extremity Impairment: A Promising Prehabilitation Strategy for Kidney Transplant Candidates. Clin Transpl (2020) 34(9):e14017. 10.1111/ctr.14017 PMC7721982 32573816

[54] Massierer D Bourgeois N Rakel A Prevost K Lands LC Poirier C Changes in 6-minute Walking Distance in Lung Transplant Candidates While Participating in a Home-Based Pre-Habilitation Program-A Retrospective Chart Review. Clin Transplant (2020) 34(10):e14045. 10.1111/ctr.14045 32686160

[55] Wickerson L Rozenberg D Helm D Gottesman C Mathur S Singer LG. Short Physical Performance Battery Scores at Lung Transplant Assessment: Relationship to Early Transplant Outcomes and Response to Pre-habilitation. J Heart Lung Transplant (2020) 39:S208–9. 10.1016/j.healun.2020.01.828 32970883

[56] Lin FP Visina JM Bloomer PM Dunn MA Josbeno DA Zhang X Prehabilitation-Driven Changes in Frailty Metrics Predict Mortality in Patients With Advanced Liver Disease. Am J Gastroenterol (2021) 116(10):2105–17. 10.14309/ajg.0000000000001376 34313620

[57] Kerti M Bohacs A Madurka I Kovats Z Gieszer B Elek J The Effectiveness of Pulmonary Rehabilitation in Connection With Lung Transplantation in Hungary. Ann (2021) 10(4):3906–15. 10.21037/apm-20-1783 33691452

[58] Layton AM Irwin AM Mihalik EC Fleisch E Keating CL Dimango EA Telerehabilitation Using Fitness Application in Patients With Severe Cystic Fibrosis Awaiting Lung Transplant: A Pilot Study. Int J Telemed Appl (2021) 2021:6641853. 10.1155/2021/6641853 33727918 PMC7935590

[59] Wickerson L Helm D Gottesman C Rozenberg D Singer LG Keshavjee S Telerehabilitation for Lung Transplant Candidates and Recipients During the COVID-19 Pandemic: Program Evaluation. JMIR MHealth and UHealth (2021) 9(6):e28708. 10.2196/28708 34048354 PMC8213059

[60] Duarte-Rojo A Bloomer PM Rogers RJ Hassan MA Dunn MA Tevar AD Introducing EL-FIT (Exercise and Liver FITness): A Smartphone App to Prehabilitate and Monitor Liver Transplant Candidates. Liver Transplant (2021) 27(4):502–12. 10.1002/lt.25950 PMC10251157 33232547

[61] Byrd R Vallabhajosula S Bailey S Champion T. Effects of Rehabilitation Before Lung Transplantation on Balance. Cardiopulmonary Phys Ther J (2022) 33(2):50–9. 10.1097/cpt.0000000000000187

[62] Singer JP Soong A Bruun A Bracha A Chin G Hays SR A Mobile Health Technology Enabled Home-Based Intervention to Treat Frailty in Adult Lung Transplant Candidates: A Pilot Study. Clin Transplant (2018) 32(6):e13274. 10.1111/ctr.13274 29742287 PMC6066279

[63] Anderson MR Easthausen I Gallagher G Udupa J Tong Y Torigian D Skeletal Muscle Adiposity and Outcomes in Candidates for Lung Transplantation: A Lung Transplant Body Composition Cohort Study. Am J Respir Crit Care Med Conf Am Thorac Soc Int Conf ATS. (2020) 201(1):A2827. 10.1164/ajrccm-conference.2020.201.1_MeetingAbstracts.A2827 PMC7888552 32482837

[64] Morkane CM Kearney O Bruce DA Melikian CN Martin DS. An Outpatient Hospital-Based Exercise Training Program for Patients With Cirrhotic Liver Disease Awaiting Transplantation: A Feasibility Trial. Transplantation (2020) 104(1):97–103. 10.1097/TP.0000000000002803 31205265

[65] Craig JA Miner D Remtulla T Miller J Zanussi LW. Piloting a Coping Skills Group Intervention to Reduce Depression and Anxiety Symptoms in Patients Awaiting Kidney or Liver Transplant. Health Soc Work (2017) 42(1):e44–e52. 10.1093/hsw/hlw064 28395080

[66] Febrero B Ramirez P Martinez-Alarcon L Abete C Galera M Rios A Group Psychotherapy Could Improve Depression in Cirrhotic Patients on the Liver Transplant Waiting List. Transpl Proc (2019) 51(1):28–32. 10.1016/j.transproceed.2018.02.206 30685106

[67] Jutagir DR Saracino RM Cunningham A Foran-Tuller KA Driscoll MA Sledge WH The Feasibility of a Group Stress Management Liver SMART Intervention for Patients With End-Stage Liver Disease: A Pilot Study. Palliat Support Care (2019) 17(1):35–41. 10.1017/S147895151800024X 29860964 PMC6279612

[68] Zhao Q Zhang S Yu R. Impact of Pre-Transplantation Psychological Counseling in Improving the Mental Well-Being of Patients on Hemodialysis. Front Psychiatr (2021) 12:594670. 10.3389/fpsyt.2021.594670 PMC7900497 33633604

[69] Zamora-Valdes D Watt KD Kellogg TA Poterucha JJ Di Cecco SR Francisco-Ziller NM Long-Term Outcomes of Patients Undergoing Simultaneous Liver Transplantation and Sleeve Gastrectomy. Hepatology (2018) 68(2):485–95. 10.1002/hep.29848 29457842

[70] Hollander FM van Pierre DD de Roos NM van de Graaf EA Iestra JA. Effects of Nutritional Status and Dietetic Interventions on Survival in Cystic Fibrosis Patients Before and After Lung Transplantation. J Cyst Fibros (2014) 13(2):212–8. 10.1016/j.jcf.2013.08.009 24041590

[71] Ju A Cazzolli R Howell M Scholes-Robertson N Wong G Jaure A. Novel Endpoints in Solid Organ Transplantation: Targeting Patient-Reported Outcome Measures. Transplantation (2023). Publish Ahead of Print. 10.1097/TP.0000000000004537 36749290

[72] Pearson N Naylor PJ Ashe MC Fernandez M Yoong SL Wolfenden L. Guidance for Conducting Feasibility and Pilot Studies for Implementation Trials. Pilot Feasibility Stud (2020) 6(1):167. 10.1186/s40814-020-00634-w 33292770 PMC7603668

[73] Perez-Saez MJ Morgado-Perez A Faura A Munoz-Redondo E Garriz M Muns MD The FRAILMar Study Protocol: Frailty in Patients With Advanced Chronic Kidney Disease Awaiting Kidney Transplantation. A Randomized Clinical Trial of Multimodal Prehabilitation. Front Med (2021) 8:675049. 10.3389/fmed.2021.675049 PMC8170320 34095178

