# Supplementary material for: European Society of Organ Transplantation (ESOT) Consensus Statement on Prehabilitation for Solid Organ Transplantation Candidates
Source: Transpl Int. 2023 Jul 21;36:11564. doi: 10.3389/ti.2023.11564 (PMC10401602; doi:10.3389/ti.2023.11564)
Supplement: Supplementary file 1 [file DataSheet2.docx]

**TLJ 3.0**

**Prehabilitation for solid organ transplant candidates**

Overview of recommendations based on the literature to discuss during the TLJ 3.0 meeting in Prague, November 13th-15th 2022.

# Background

For solid organ transplant candidates (SOTCs) it is important to be in an optimal condition, both physically and psychologically, to be able to endure the stress of transplant surgery and to enhance recovery after the transplant. However, the overall fitness of transplant candidates is often compromised due to end-stage organ failure, comorbidities, and, in case of chronic kidney disease, adverse effects of dialysis. The overall condition of SOTCs is often characterized by low physical activity, malnutrition, and fatigue, which in turn may cause psychological problems. Consequently, SOTCs may be considered as a frail patient population. The waiting-list period before a solid organ transplant provides a window of opportunity to enhance the overall condition of SOTCs by prehabilitation.

Prehabilitation refers to the process of optimizing the overall condition of a patient before an operation in order to enhance their ability to withstand the stress of the surgery and accelerate recovery after surgery.[1] Prehabilitation focuses on achieving lifestyle changes in order to reduce risks of complications related to surgery and should at least comprise physical training, dietary management, and psychological interventions.[1]

Prehabilitation has shown promising results in other patient populations [2-4] and may also be beneficial for SOTCs.[5, 6] By offering prehabilitation prior to transplantation, patients may be more likely to adopt a sustainable, healthy lifestyle. Therefore, prehabilitation may not only improve the health and quality of life of SOTCs before and after the transplant, but may improve clinical outcomes, e.g. a lower complication rate, a shorter length of hospital stay and improved survival rates.

Studies investigating prehabilitation in transplant populations are scarce. So far, a few studies have shown that prehabilitation during the waiting-list period is feasible.[5, 6] Consequently, recommendations which exercise, nutritional of psychological interventions are effective and suitable for prehabilitation in SOTCs are lacking nor a guideline for prehabilitation in SOTCs has not been established.

To gain insight into the evidence for prehabilitation interventions in SOTCs, a literature review was performed based on the following questions:

1. In solid organ transplant candidates, what is the evidence for exercise training/nutritional support/ psychosocial interventions pre-transplant?
2. In solid organ transplant candidates, what type(s) of exercises/nutritional support/psychosocial interventions are recommended in the pre-transplant phase?
3. In solid organ transplant candidates, what are the outcomes relevant to exercise and physical activity/nutritional support/psychosocial interventions that should be measured pre-transplant?
4. In solid organ transplant candidates, what is the evidence for the feasibility of prehabilitation?

Hereby we provide the recommendations for prehabilitation of SOTCs developed during the ESOT Transplant Learning Journey 3.0 conference held in Prague, Czech Republic, on November 13-15, 2022.

# **Recommendations Physical exercise interventions**

Q1 In SOTC, what is the evidence for exercise training pre-transplant Q3 Expected outcomes relevant to exercise in the pre-transplant phase

|  | Deliberations | Level of evidence* |
| --- | --- | --- |
| 1 | **Overall,** evidence on the effectiveness of exercise interventions prior to solid organ transplantation remains inconclusive, given the limited number of RCTs (n=7 unique studies) and evaluated patients (n=198), the heterogeneity in the study population (94 lung transplant candidates, 67 heart transplant candidates, 37 liver transplant candidates, and 0 kidney transplant candidates), the variety of outcomes and interventions being considered, the overall moderate methodological quality, and the use of small sample sizes by the published studies.[7-14] | VERY LOW |
| 2 | Evidence suggests that   1. Heart transplant candidates may benefit from exercise interventions to increase their cardiorespiratory fitness (VO2peak and/or 6MWD), with clinical meaningful effect size, although the number of large RCTs is limited.[7, 10-12] 2. Heart, lung, and liver transplant candidates might benefit from exercise interventions to increase their inspiratory muscle strength, with clinical meaningful effect size, although the number of large randomized controlled studies is limited.[9, 11, 13, 14] 3. The lack of RCTs did not allow meta-analyses to be performed for other outcomes | LOW |
| 3 | Most studies include cardiorespiratory fitness (VO2peak and 6MWD), Health related Quality of Life, dyspnoea, or maximal inspiratory pressure as an outcome measure.  Other markers of health-related physical fitness [18] remain to be investigated:   - Muscular fitness (e.g., muscle strength, muscle endurance, muscle power), - motor fitness (e.g., balance, coordination, agility, speed of movement), - body morphology (e.g., muscle and fat content, bone density), - (cardio)metabolic health.   Other critically important but yet to be investigated outcomes are:   - Patient-reported outcomes: fatigue, activities of daily living - Clinical outcomes: frailty, mortality on the waiting list, mortality after transplantation, complications, length of ICU and hospital stay, hospital (re)admissions, complications, and graft function and survival. | NA |

*HIGH=we are very confident that the true effect lies close to the estimate of the effect. MODERATE= we are moderately confident in the effect estimate: The true effect is likely to be close to the estimate of the effect, but there is a possibility that it is substantially different. LOW= Our confidence in the effect estimate is limited: The true effect may be substantially different from the estimate of the effect. VERY LOW= We have very little confidence in the effect estimate: The true effect is likely to be substantially different from the estimate of effect. NA=not applicable

### **Recommendations**

We recommend that further studies are needed to evaluate the effectiveness of exercise-based prehabilitation in all types of solid organ transplant candidates.

There is some evidence to suggest that exercise training increases cardiorespiratory fitness in heart transplant candidates, and that it increases inspiratory muscle strength in heart, lung and liver transplant candidates.

In order to make further progress in this field, we strongly recommend that a Core Outcome Measurement Set is defined for future exercise-based prehabilitation studies for solid organ transplant candidates.

## Q2. Type(s) of exercises recommended in the pre-transplant phase

|  | Deliberations | Level of evidence |
| --- | --- | --- |
| 1 | Most studies use aerobic training [7, 8, 10-12], peripheral muscle training [16], inspiratory muscle strength training [9, 17], or a combination of these training modalities. However, the optimal characteristics of the exercise interventions regarding Frequency, Intensity, Time, and Type (FITT principle) remains unknown. | VERY LOW |
| 2 | The optimal mode of delivery (home-based, inpatient, outpatient, and level of supervision) remains unknown. | VERY LOW |

*HIGH=we are very confident that the true effect lies close to the estimate of the effect. MODERATE= we are moderately confident in the effect estimate: The true effect is likely to be close to the estimate of the effect, but there is a possibility that it is substantially different. LOW= Our confidence in the effect estimate is limited: The true effect may be substantially different from the estimate of the effect. VERY LOW= We have very little confidence in the effect estimate: The true effect is likely to be substantially different from the estimate of effect. NA=not applicable

### **Recommendations**

We recommend that further research is needed to identify the optimal characteristics and mode of delivery of exercise-based prehabilitation for solid organ transplant candidates. Future research should also focus on the effectiveness of supervised in-person exercise training compared to home-based or virtual prehabilitation.

We suggest that characteristics and mode of delivery of exercise training should be tailored to the individual needs and capabilities of every transplant candidate.

We recommend that balance and flexibility training should be investigated in future research as these have not been studied in solid transplant candidates so far.

## Q4. Evidence for the feasibility of prehabilitation regarding exercise intervention

|  | **Deliberations** | **Level of evidence*** |
| --- | --- | --- |
| 1 | Studies that specifically focused on feasibility of exercise-based prehabilitation intervention are lacking. | NA |
| 2 | In total, 7 RCTs described one or more aspects of feasibility:   - Reach (how many patients accepted to participate in the study out of those who were offered), was about 86%. - protocol adherence: insufficiently reported to draw conclusions - training compliance: insufficiently reported to draw conclusions - Drop-out rate: within the context of the pretransplantation setting, the observed average dropout rate of 20% in the exercise-based prehabilitation group and 11% in the control group is considered low to moderate. | Very Low |

**HIGH=we are very confident that the true effect lies close to the estimate of the effect. MODERATE= we are moderately confident in the effect estimate: The true effect is likely to be close to the estimate of the effect, but there is a possibility that it is substantially different. LOW= Our confidence in the effect estimate is limited: The true effect may be substantially different from the estimate of the effect. VERY LOW= We have very little confidence in the effect estimate: The true effect is likely to be substantially different from the estimate of effect. NA=not applicable

### **Recommendations**

The limited evidence available suggests that it may be feasible to provide exercise-based prehabilitation in solid organ transplant candidates. Whether different subpopulations might need specific training content needs to be addressed.

Further studies are needed to gain insight into the feasibility of various components of exercise-based prehabilitation (e.g., frequency, intensity, time, type), and delivery mode (in-person, centre based training vs home-based vs virtual training).

Strategies to promote implementation (acceptability, reach, protocol adherence, training compliance how to retain participants) of exercise-based prehabilitation in the clinical setting should be identified in future studies.

# **Recommendations Nutritional interventions**

## Q1. In SOTC, what is the evidence for interventions pre-transplant?

|  | Deliberations | Level of evidence* |
| --- | --- | --- |
| 1 | Overall, evidence on the efficacy of nutritional interventions prior to solid organ transplantation remains inconclusive, given the limited number of studies (n=7)[19-25] the variety of outcomes and interventions being considered, the inconsistency of findings across studies, the overall low methodological quality and the use of small sample sizes by the majority of the published studies. | Very low |
| 2 | Evidence [21-25] suggests that SOTCs might benefit from nutritional intervention to either improve energy intake (lower or higher) or reduce weight, but effect size is small and the number of studies is limited. | Very Low |
| 3 | Evidence [19, 20]suggests that SOTCs might benefit from probiotic therapy to reduce post liver transplant infections. | Medium |
| 3 | The effect of nutritional interventions on hard medical outcomes (survival) was examined as a secondary outcome in 4 studies [19-22, 25] and showed no significant effect of the intervention on survival before or after the transplant. | Very low |
| 4 | Because of the heterogeneity in the content of the nutritional interventions and the mode of delivery, it remains unclear how the intervention is best delivered. [19-25] | Very low |

*HIGH=we are very confident that the true effect lies close to the estimate of the effect. MODERATE= we are moderately confident in the effect estimate: The true effect is likely to be close to the estimate of the effect, but there is a possibility that it is substantially different. LOW= Our confidence in the effect estimate is limited: The true effect may be substantially different from the estimate of the effect. VERY LOW= We have very little confidence in the effect estimate: The true effect is likely to be substantially different from the estimate of effect. NA=not applicable

### **Recommendations**

We recommended that further studies are needed to evaluate the effectiveness of nutritional interventions in solid organ transplant candidates. There is some evidence to suggest the use of probiotic therapy to reduce post-transplant infections. We recommend further research into nutritional interventions pre-transplant with a pre-defined Core Outcome Measurement Set.

**Q2. In SOTC, what type of interventions are recommended pre-transplant?**

|  | Deliberations | Level of evidence* |
| --- | --- | --- |
| 1 | Most studies [21-25] use either nutritional support to aid energy intake (increase or decrease) or a weight loss intervention to prepare SOT candidates for surgery. | Very low |
| 2 | Based on the evidence from 2 intervention studies [21, 25] and 1 retrospective study [22], providing increased energy intake before transplantation may allow target weight to be reached, especially in the underweight patients. | Very low |
| 3 | For patients with overweight it is recommended to obtain the involvement of a dietician for a weight loss programme [23]. | Low |
| 4 | It is suggested to use probiotics before liver transplantation to reduce post-transplant infection rates. [19, 20] | Low |
| 3 | It is suggested that perioperative immunonutrition (n-3 fatty acids, arginine, and nucleotides) does not improve preoperative nutritional status or postoperative outcome. [24] | Low |

*HIGH=we are very confident that the true effect lies close to the estimate of the effect. MODERATE= we are moderately confident in the effect estimate: The true effect is likely to be close to the estimate of the effect, but there is a possibility that it is substantially different. LOW= Our confidence in the effect estimate is limited: The true effect may be substantially different from the estimate of the effect. VERY LOW= We have very little confidence in the effect estimate: The true effect is likely to be substantially different from the estimate of effect. NA=not applicable

### **Recommendations**

We recommend that further research is needed to examine the optimal characteristics and mode of delivery of nutritional interventions for solid organ transplant candidates.

It is suggested that interventions are utilised to increase energy intake for SOTCs with underweight. Interventions might be considered when aiming to reduce weight pre-transplant. If bariatric surgery is not offered, then it is suggested that a healthcare professional is involved to deliver weight loss interventions.

The use of probiotics might be promising to prepare candidates for liver transplantation, but further research is needed to demonstrate their effectiveness in other SOT candidates.

## **Q3. In SOTC, what are the relevant outcomes that should be measured pre- and posttransplant?**

|  | **Deliberations** | **Level of evidence*** |
| --- | --- | --- |
| 1 | Most studies included weight change or weight loss as an outcome measure. [21-25] | NA |
| 2 | Two studies [19, 20] included post-transplant infection rate as a primary outcome measure | NA |
| 3 | In addition, total body protein, nutritional status, fat mass, 6-minute walk test distance, hand grip strength and survival were measured as secondary outcomes across studies. | NA |
| 4 | The studies included in this review varied in methods to assess these outcomes. | NA |

*HIGH=we are very confident that the true effect lies close to the estimate of the effect. MODERATE= we are moderately confident in the effect estimate: The true effect is likely to be close to the estimate of the effect, but there is a possibility that it is substantially different. LOW= Our confidence in the effect estimate is limited: The true effect may be substantially different from the estimate of the effect. VERY LOW= We have very little confidence in the effect estimate: The true effect is likely to be substantially different from the estimate of effect. NA=not applicable

### **Recommendations**

We suggest that weight and body composition should be considered as an outcome in prehabilitation studies for SOTC as this is relatively easy to measure. Infection rate may be a useful outcome to assess the effect of probiotic therapy.

We suggest that a Core Outcome Measurement Set should be defined for future prehabilitation nutritional intervention studies for SOTC.

## Q4 In SOTC, what is the evidence for the feasibility of prehabilitation?

|  | **Deliberations** | **Level of evidence*** |
| --- | --- | --- |
| 1 | No studies were designed to specifically assess feasibility of nutritional interventions in prehabilitation care for SOTC. | Very low |
| 2 | Only one study closed early due to acceptability of trial procedures. Attrition rate in remaining 6 studies was low. | Very Low |

*HIGH=we are very confident that the true effect lies close to the estimate of the effect. MODERATE= we are moderately confident in the effect estimate: The true effect is likely to be close to the estimate of the effect, but there is a possibility that it is substantially different. LOW= Our confidence in the effect estimate is limited: The true effect may be substantially different from the estimate of the effect. VERY LOW= We have very little confidence in the effect estimate: The true effect is likely to be substantially different from the estimate of effect. NA=not applicable

**Recommendations**

Although there is limited evidence available that directly measures feasibility of delivering nutritional interventions, we suggest that it is feasible to provide nutritional interventions for prehabilitation in SOTCs, particularly where there is a healthcare professional present in the clinic.

We suggest that SOTCs might benefit from seeing a healthcare professional to assess and intervene with nutritional interventions prior to transplantation focusing on improving nutritional status and energy intake of candidates with underweight, reduce weight prior to transplantation in SOTCs with overweight, or potentially prescribe probiotic therapy.

# **Recommendations** **psychosocial interventions**

## Q1. In SOTC, what is the evidence for interventions pre-transplant?

|  | Deliberations | Level of evidence* |
| --- | --- | --- |
| 1 | Overall, evidence on the effectiveness of psychosocial interventions prior to solid organ transplantation remains inconclusive, given the limited number of studies (n=10), the variety of outcomes and interventions being considered, the inconsistency of findings across studies, the overall low methodological quality and the use of small sample sizes by the majority of the published studies.[26-35] | VERY LOW |
| 2 | Evidence suggests that SOTCs might benefit from psychosocial intervention to reduce their level of anxiety, depression and/or fatigue or improve their quality of life, but effect size is small and the number of studies is limited. [26, 31, 33, 35] | LOW |
| 3 | The effect of psychosocial interventions on hard medical outcomes (survival, readmissions) was examined in only one study, showing no significant effect of the intervention on survival before or after the transplant.[31] | VERY LOW |
| 4 | Because of the heterogeneity in the content of the psychosocial interventions and the mode of delivery, it remains unclear how the intervention is best delivered (e.g. face-to-face vs telephone, individual vs group), by whom (e.g. nurses, psychologists, others)), the frequency (e.g. weekly, bi-weekly) and the duration (1 to 6 months). [26-35] | VERY LOW |

*HIGH=we are very confident that the true effect lies close to the estimate of the effect. MODERATE= we are moderately confident in the effect estimate: The true effect is likely to be close to the estimate of the effect, but there is a possibility that it is substantially different. LOW= Our confidence in the effect estimate is limited: The true effect may be substantially different from the estimate of the effect. VERY LOW= We have very little confidence in the effect estimate: The true effect is likely to be substantially different from the estimate of effect. NA=not applicable

### **Recommendations**

We recommended that further studies are needed to evaluate the effectiveness of psychosocial interventions in solid organ transplant candidates in which the interventions are compared with usual care

## Q2. In SOTC, what type of interventions are recommended pre-transplant?

|  | Deliberations | Level of evidence* |
| --- | --- | --- |
| 1 | Most studies use a combination of cognitive behavioural therapy, psycho-educational interventions and relaxation techniques. [26-35] | VERY LOW |
| 2 | The content of the psychosocial interventions and the mode of delivery, it remains unclear how the intervention is best delivered (e.g. face-to-face vs telephone, individual vs group), by whom (e.g. nurses, psychologists, others)), the frequency (e.g. weekly, bi-weekly) and the duration (1 to 6 months). [26-35] | VERY LOW |
| 3 | Cognitive behavioural therapy and psychoeducational interventions might be effective to reduce anxiety and depression. [31, 33] | LOW |

*HIGH=we are very confident that the true effect lies close to the estimate of the effect. MODERATE= we are moderately confident in the effect estimate: The true effect is likely to be close to the estimate of the effect, but there is a possibility that it is substantially different. LOW= Our confidence in the effect estimate is limited: The true effect may be substantially different from the estimate of the effect. VERY LOW= We have very little confidence in the effect estimate: The true effect is likely to be substantially different from the estimate of effect. NA=not applicable

### **Recommendations**

We recommend that further research is needed to examine the optimal content and mode of delivery of psychosocial interventions for solid organ transplant candidates.

It is suggested that cognitive behavioural therapy and psychoeducational interventions might be considered when aiming to reduce symptoms of anxiety and depression in solid organ transplant candidates.

Stress-reducing interventions, such as mindfulness based stress reduction or relaxation techniques, might be promising to reduce anxiety or stress levels, but further research is needed to demonstrate their effectiveness.

## Q3. In SOTC, what are the relevant outcomes that should be measured pre-transplant?

|  | **Deliberations** | **Level of evidence*** |
| --- | --- | --- |
| 1 | Most studies included Health Related Quality of Life (HRQoL) as an outcome measure. [26, 29-31, 33, 35] | NA |
| 2 | In addition, depression, anxiety, fatigue, sleep quality, social intimacy and coping skills were measured as outcomes. [26-35] | NA |
| 3 | The studies included in this review varied in methods to assess these outcomes. [26-35] | NA |
| 4 | Clinical outcomes were only considered in one study. [31] | NA |

*HIGH=we are very confident that the true effect lies close to the estimate of the effect. MODERATE= we are moderately confident in the effect estimate: The true effect is likely to be close to the estimate of the effect, but there is a possibility that it is substantially different. LOW= Our confidence in the effect estimate is limited: The true effect may be substantially different from the estimate of the effect. VERY LOW= We have very little confidence in the effect estimate: The true effect is likely to be substantially different from the estimate of effect. NA=not applicable

### **Recommendations**

We suggest that health related quality of life should be considered as an outcome in studies on prehabilitation of SOTC.

We suggest that outcomes measured in studies on psychosocial interventions among SOTCs should comprise of a measure of distress, anxiety and/or depression symptoms.

We suggest that solid organ transplant candidates might benefit from interventions prior to transplantation focusing on improving their overall health-related quality of life and alleviating symptoms of depression, stress, anxiety or fatigue. Yet, other outcomes that are potentially of relevance during the pre-Tx waiting time are not yet considered (such as medication adherence or lifestyle issues), nor did studies investigate the impact on medical outcomes (such as survival on the waiting list or post-Tx morbidity and mortality).

We suggest that a Core Outcome Measurement Set should be defined for future prehabilitation psychosocial intervention studies for SOTC.

## Q4 In SOTC, what is the evidence for the feasibility of prehabilitation?

|  | **Deliberations** | **Level of evidence*** |
| --- | --- | --- |
| 1 | Only one study specifically focused on feasibility of a prehabilitation psychological intervention. [27] | C |
| 2 | In total, seven studies described one aspect of feasibility   - Enrolment: varied between 24%-59% [27, 30, 31] - Attendance rate: 69%-88% [26, 27, 31] - Satisfaction with the intervention: 90-100% [26, 27] - Drop-out: overall moderate drop-out rates 9%-18%. [26, 27, 29-31, 33] In one study the drop-out was high 53%. [34] | D |
| 3 | Three studies described that the participants found the intervention to be supportive and helpful [27, 28, 30] | D |

*HIGH=we are very confident that the true effect lies close to the estimate of the effect. MODERATE= we are moderately confident in the effect estimate: The true effect is likely to be close to the estimate of the effect, but there is a possibility that it is substantially different. LOW= Our confidence in the effect estimate is limited: The true effect may be substantially different from the estimate of the effect. VERY LOW= We have very little confidence in the effect estimate: The true effect is likely to be substantially different from the estimate of effect. NA=not applicable

### **Recommendations**

The limited evidence available suggests that it might be feasible to provide psychosocial interventions for prehabilitation in SOTCs.

Further studies are needed to gain insight into specific aspects exploring the feasibility of psychosocial interventions for SOTCs regarding enrolment (whom needs which type of care), how to reach the target group, how to retain participants, how to encourage intervention adherence and the feasibility of its implementation in daily practice.

# **References**

1. Minnella, E.M. and F. Carli, *Prehabilitation and functional recovery for colorectal cancer patients.* Ejso, 2018. **44**(7): p. 919-926.

2. Heil, T.C., et al., *Improved Postoperative Outcomes after Prehabilitation for Colorectal Cancer Surgery in Older Patients: An Emulated Target Trial.* Ann Surg Oncol, 2022.

3. Granicher, P., et al., *Prehabilitation Improves Knee Functioning Before and Within the First Year After Total Knee Arthroplasty: A Systematic Review With Meta-analysis.* J Orthop Sports Phys Ther, 2022. **52**(11): p. 709-725.

4. van Wijk, L., et al., *Improved preoperative aerobic fitness following a home-based bimodal prehabilitation programme in high-risk patients scheduled for liver or pancreatic resection.* Br J Surg, 2022. **109**(11): p. 1036-1039.

5. Wallen, M.P., et al., *Safety, adherence and efficacy of exercise training in solid-organ transplant candidates: A systematic review.* Transplantation Reviews, 2016. **30**(4): p. 218-26.

6. Pesce de Souza, F., et al., *Exercise interventions in solid organ transplant candidates: a systematic review.* Clinical Transplantation, 2020. **34**(9): p. e13900.

7. Laoutaris, I.D., et al., *Benefits of physical training on exercise capacity, inspiratory muscle function, and quality of life in patients with ventricular assist devices long-term postimplantation.* European Journal of Cardiovascular Prevention & Rehabilitation, 2011. **18**(1): p. 33-40.

8. Gloeckl, R., M. Halle, and K. Kenn, *Interval versus continuous training in lung transplant candidates: A randomized trial.* The Journal of Heart and Lung Transplantation, 2012. **31**(9): p. 934-41.

9. Pehlivan, E., et al., *The effects of inspiratory muscle training on exercise capacity, dyspnea and respiratory functions in lung transplantation candidates: a randomized controlled trial.* Clinical Rehabilitation, 2018. **32**(10): p. 1328-1339.

10. Adamopoulos, S., et al., *Thyroid hormone signalling is altered in response to physical training in patients with end-stage heart failure and mechanical assist devices: potential physiological consequences?* Interactive Cardiovascular & Thoracic Surgery, 2013. **17**(4): p. 664-8.

11. Forestieri, P., et al., *A Cycle Ergometer Exercise Program Improves Exercise Capacity and Inspiratory Muscle Function in Hospitalized Patients Awaiting Heart Transplantation: a Pilot Study.* Brazilian Journal of Cardiovascular Surgery, 2016. **31**(5): p. 389-395.

12. Hayes, K., et al., *Effects of exercise training on exercise capacity and quality of life in patients with a left ventricular assist device: A preliminary randomized controlled trial.* Journal of Heart and Lung Transplantation, 2012. **31**(7): p. 729-734.

13. Limongi, V., et al., *Effects of a respiratory physiotherapeutic program in liver transplantation candidates.* Transplantation Proceedings, 2014. **46**(6): p. 1775-7.

14. Limongi, V., et al., *Exercise manual for liver disease patients.* World J Transplant, 2016. **6**(2): p. 429-36.

15. Florian, J., et al., *Pulmonary rehabilitation improves survival in patients with idiopathic pulmonary fibrosis undergoing lung transplantation.* Scientific Reports, 2019. **9**.

16. Fuller, L.M., et al., *Running with a VAD.* Journal of Heart and Lung Transplantation, 2014. **1)**: p. S222.

17. Kilic, L., et al., *Effect of 8-week pulmonary rehabilitation program on dyspnea and functional capacity of patients on waiting list for lung transplantation.* Turkish Thoracic Journal, 2020. **21(2)**: p. 110-115.

18. Pelliccia, A., et al., *ESC 2020 Guideline on sport and exercise cardiology in patients with cardiovascular disease ESC task force on sport cardiology and exercise cardiology in patients with cardiovascular disease.* Revista Espanola De Cardiologia, 2021. **74**(6).

19. Eguchi, S., et al., *Perioperative synbiotic treatment to prevent infectious complications in patients after elective living donor liver transplantation: a prospective randomized study.* Am J Surg, 2011. **201**(4): p. 498-502.

20. Grat, M., et al., *Effects of continuous use of probiotics before liver transplantation: A randomized, double-blind, placebo-controlled trial.* Clinical Nutrition, 2017. **36**(6): p. 1530-1539.

21. Forli, L., et al., *A study of intensified dietary support in underweight candidates for lung transplantation.* Annals of Nutrition & Metabolism, 2001. **45**(4): p. 159-68.

22. Forli, L., et al., *Dietary support to underweight patients with end-stage pulmonary disease assessed for lung transplantation.* Respiration, 2001. **68**(1): p. 51-7.

23. Park, T.L., M.G. Perri, and J.R. Rodrigue, *Minimal intervention programs for weight loss in heart transplant candidates: a preliminary examination.* Progress in Transplantation, 2003. **13**(4): p. 284-8.

24. Plank, L.D., et al., *Perioperative immunonutrition in patients undergoing liver transplantation: a randomized double-blind trial.* Hepatology, 2015. **61**(2): p. 639-47.

25. Le Cornu, K.A., et al., *A prospective randomized study of preoperative nutritional supplementation in patients awaiting elective orthotopic liver transplantation.* Transplantation, 2000. **69**(7): p. 1364-9.

26. Gross, C.R., et al., *Telephone-adapted Mindfulness-based Stress Reduction (tMBSR) for patients awaiting kidney transplantation.* Contemporary Clinical Trials, 2017.

27. Jutagir, D.R., et al., *The feasibility of a group stress management Liver SMART intervention for patients with end-stage liver disease: A pilot study.* Palliative & Supportive Care, 2019. **17**(1): p. 35-41.

28. Craig, J.A., et al., *Piloting a Coping Skills Group Intervention to Reduce Depression and Anxiety Symptoms in Patients Awaiting Kidney or Liver Transplant.* Health & Social Work, 2017. **42**(1): p. e44-e52.

29. Rodrigue, J.R., et al., *A randomized evaluation of quality-of-life therapy with patients awaiting lung transplantation.* American Journal of Transplantation, 2005. **5**(10): p. 2425-32.

30. Rodrigue, J.R., D.A. Mandelbrot, and M. Pavlakis, *A psychological intervention to improve quality of life and reduce psychological distress in adults awaiting kidney transplantation.* Nephrology Dialysis Transplantation, 2011. **26**(2): p. 709-15.

31. Blumenthal, J.A., et al., *Telephone-based coping skills training for patients awaiting lung transplantation.* J Consult Clin Psychol, 2006. **74**(3): p. 535-44.

32. Zhao, Q., S. Zhang, and R. Yu, *Impact of Pre-Transplantation Psychological Counseling in Improving the Mental Well-Being of Patients on Hemodialysis.* Frontiers in psychiatry Frontiers Research Foundation, 2021. **12**: p. 594670.

33. Napolitano, M.A., et al., *Effects of a telephone-based psychosocial intervention for patients awaiting lung transplantation.* Chest, 2002. **122**(4): p. 1176-84.

34. Febrero, B., et al., *Group Psychotherapy Could Improve Depression in Cirrhotic Patients on the Liver Transplant Waiting List.* Transplantation Proceedings, 2019. **51**(1): p. 28-32.

35. Sharif, F., et al., *Effects of psycho-educational intervention on health-related quality of life (QOL) of patients with chronic liver disease referring to Shiraz University of Medical Sciences.* Health Qual Life Outcomes, 2005. **3**: p. 81.
