# Supplementary material for: European Society of Organ Transplantation (ESOT) Consensus Statement on Prehabilitation for Solid Organ Transplantation Candidates
Source: Transpl Int. 2023 Jul 21;36:11564. doi: 10.3389/ti.2023.11564 (PMC10401602; doi:10.3389/ti.2023.11564)
Supplement: Supplementary file 2 [file DataSheet1.docx]

# **Supplementary file 1: Summary of non-RCTs- exercise & psychosocial interventions**

|  | **First author, year (country of origin)** | **Sample characteristics& study design**  Tx-type, design, n per group, % male, age (y) (mean (sd) or median (range) | **Intervention(s)** | **Effectiveness outcomes** | **Results – Effectiveness outcomes**  ↑ = significant increase  ↓ = significant decrease  ≈ no difference | **Results – Feasibility outcomes** |
| --- | --- | --- | --- | --- | --- | --- |
| **Exercise interventions** | | | | | | |
| **1** | Ben-Gal, 2000 (Israel) | HTx candidates  Design: Prospective cohort study that finalized as case-control  Total n=12  **I**: n=6  % male: NR  Age: NR  **C**: n=6  % male: NR  Age: NR | **I:** 6.5 months, hospital-based, aerobic interval training with cycle ergometer (cycling: 15 min/session, intensity 30 s at 50% of Wmax alternated by 60 s of recovery at 10-20 W) + and treadmill walking (duration NR, 2x/week, intensity 60 s slow walking (10/6-20 Borg scale) alternated by 60 s fast walking (14/6-20 Borg scale)) supervised by physician  **C:** No training | VO_2_peak  VO_2_ at anaerobic threshold  W_max_  6MWD  **Assessments:**  - Before intervention  - After intervention | **VO_2_peak:** ↑ within I; ↓ within C  **VO_2_ at anaerobic threshold**: ↑ within I; ↓ within C  **W_max_**: ↑ within I; ↓ within C  **6MWD**: ↑ within I | **Enrolment**: n=18 assessed for eligibility, n= 6 (33%) were incapable to participate in the intervention  **Attrition**: 10/12 (83%), I: 5/6 (83%); C : 5/6 (83%). Drop outs due to HTx (n=1 in both groups)  **Acceptability**: 2 patients did not ‘tolerate’ the training intervention, 4 patients soon stopped training due to geographical inconvenience  **Fidelity** (participants): 92% of the exercise sessions were attended  **Fidelity** (interventionist): NR  **Safety**: No adverse events (significant cardiovascular event) occurred |
| **2** | Karapolat, 2013 (Turkey) | HTx candidates  Design: Retrospective pre-post cohort study  **I**: n=11  86% male  Age: 46 (±14) | **I:** 8-weeks, supervised, hospital-based, flexibility, strength, aerobic exercises, breathing exercises, and relaxation exercises (90 min session, 3x/week; intensity: Aerobic training: 60-70% VO_2_peak, 12-14/6-20 Borg scale; strength training: 250-500 g weight for training of upper and lower extremities) | VO_2_peak  Pulmonary function (FEV1, FVC, FEV1/FVC)  Psychological well-being: depression (BDI) and anxiety (STAI)  QOL (SF36)  **Assessments:**  - Before intervention  - After intervention | **VO_2_peak:** ↑  **FEV1:** ↑  **FVC**: ↑  **FEV1/FVC**: ≈  **BDI**: ↑  **STAI** : ≈  **SF36**: ↑ in most subscales | **Enrolment**: NR  **Attrition**: NR  **Acceptability**: NR  **Fidelity** (participants): NR  **Fidelity** (interventionist): NR  **Safety**: NR |
| **3** | Florian, 2013 (Brazil) | LuTx candidates  Design: Prospective study  **I:** n=58  48% male  Age: 46 (±14) | **I:** 36-weeks, supervised by physical therapists, strength training comprised of warmups, breathing exercises associated with arm raising, arm and leg muscle strengthening ( 90 min/session, 3x/week, intensity: start at 30% 1RM, 1 set of 10 repetitions per exercise. Load was increased by 0.5 kg every 7 sessions to patient tolerance) + aerobic training: treadmill (30 min/ session, 3x/week; intensity: start at 60% 6MWT speed, with progressive protocol every 6 minutes for the variable time until reaching 30 minutes. Speed was increased by 0.3 km/h every 7 sessions)  In addition clinical evaluation, psychiatric evaluation, nutritional counselling, social assistance, and educational lectures were provided | 6MWD  QOL (SF36)  Dyspnea at rest and after exercise  Leg fatigue at rest and after exercise  SpO_2_ at rest and after exercise  **Assessments:**  - Before intervention  - After intervention | **6MWD**: ↑ (+ 72m)  **SF36:** ↑ in physical functioning, role-physical, bodily pain, vitality, social functioning, and mental health. **≈** in general health and role-emotional  **Dyspnea at rest**: ↓  **Dyspnea after exercise**: ↓  **Leg fatigue at rest**: ≈  **Leg fatigue after exercise**: ↓  **SpO_2_ at rest and after exercise**: **≈** | **Enrolment**: 112 referred for rehabilitation and included  **Attrition**: 58/112 (52%) Dropouts due to LuTx (n=43), death (n=8), gave up LuTx (n=2), long hospitalization (n=1)  **Acceptability**: NR  **Fidelity** (participants): NR  **Fidelity** (interventionist): NR  **Safety**: NR |
| **4** | Li, 2013 (Canada) | LuTx candidates  Design: Retrospective cohort study  **I:** n=345  55% male  Age: 51 (±14) | **I:** entire waiting period and first 3 months after Tx, supervised by physical therapists/ physical therapy assistant, aerobic (treadmill, cycle, and arm ergometer) + strength + stretching exercises (1.5 to 2 hours/session, 3x/week, intensity based on patient’s symptoms, heart rate, oxygen saturation and rating of perceived exertion) | 6MWD  QOL (5 different instruments)  Exercise training details  **Assessments:**  - Time of listing  - 6 weeks  - 12 weeks  - Every 3 months until LuTx | **6MWD**: ↓; 6MWD prior to LuTx was 15 m less than 6MWD at listing  **QOL**: ↓  **Exercise training volumes:** ↑ | **Enrolment**: 422/435 (97%) eligible  **Attrition**: 345/422 (82%) participants with complete data  **Acceptability**: NR  **Fidelity** (participants): NR  **Fidelity** (interventionist): NR  **Safety**: NR |
| **5** | Debette-Gratien, 2015 (France) | LiTx candidates  Design: Prospective pre-post cohort study  **I:** n=13  75% male  Age: 51 (±12) | **I:** 12-weeks, hospital based, supervised by nurse and physician, adapted aerobic (cycling) + strength training (120 min/session, 2x/week, intensity aerobic training (cycling) at ventilatory threshold power for at least 20 min; strength training: 3 sets of 8-13 repetitions at 70-80% 1RM) | Acceptability  VO_2_peak  W_max_  Power at ventilatory threshold  6MWD  Knee extensor muscle strength  QOL ( SF36)  **Assessments:**  - Before intervention  - After intervention | **VO_2_peak:** ↑  **W_max_:** ↑  **Power at ventilatory threshold:** ↑  **6MWD:** ↑  **Knee extensor muscle strength:** ↑  **SF36:** ≈ | **Enrolment**: NR  **Attrition**: 8/13 (62%); Dropouts (n=5) due to: moved to other region (n=2), LiTx (n=2), deteriorated clinical condition (n=1)  **Acceptability**: one patient interrupted the study because of health reasons  **Fidelity** (participants): n=4 interrupted study due to logistics; n= 2 interrupted study for personal convenience  **Fidelity** (interventionist): NR  **Safety**: No adverse events (cardiovascular events or cirrhosis decompensation) were observed |
| **6** | Kenn, 2015 (Germany) | LuTx candidates  Design: Retrospective cohort study  Total group (n=811)  comprised of  4 subgroups  **I1:** n=360 LuTxc/COPD  41% male  Age: 54 (±7.6)  **I2**: n=127  LuTxc/AATP  17% male  Age: 51 (±6)  **I3** n=195  LuTxc/ILD  27% male  Age: 54 (±8.7)  **I4** n=69  LUtxc/CF  11% male  Age: 53 (±7.4) | **I**: 5 weeks, supervised, aerobic (endurance training, 10 to 20 min/session, 5-6x/week, intensity 60% of peak work rate) + strength training (30 to 45 min/session, 5-6x/week, intensity: 4-6 exercises, 3x 20 repetitions per exercise at the maximum tolerated load) | 6MWD  QOL (SF36)  **Assessments:**  - Before intervention  - After intervention | **6MWD:** ↑ (+55.9 ± 58.3 m)  **SF36** : ↑ in physical (+1.9 ± 8.5) and mental (+8.7 ± 13.5) component scores | **Enrolment**: All LuTx candidates referred for inpatient rehabilitation with complete pre-test and post-test data for 6MWT; n=811  **Attrition**: NR  **Acceptability**: NR  **Fidelity** (participants): NR  **Fidelity** (interventionist): NR  **Safety**: NR |
| **7** | Singer, 2018 (United States) | LuTx candidates  Design: Prospective study  **I:** n=15  67% male  Age: 63 (±6) | **I**: 8 weeks home-based exercise training guided by weekly telephone check-ins (goal setting, barriers identification, support, positive reinforcement, and feedback using motivational interviewing techniques) and a mobile device application linked with a Fitbit activity tracker. Exercise included walking, sit to stands, tandem walking, wall push-ups, pursed-lip breathing, and stretching. Aerobic exercise was initiated at 65-75% of participants’ exercise capacity estimated by 6MWD. Intensity of strength exercises (elastic bands) was based on participants’ SPPB score and grip strength. | Safety  Feasibility  Frailty (SPPB  And FFP)  6MWD  Grip strength  Self-reported disability and functional capacity (DASI and LT-VLA) | **Frailty**: trend for improvement  **6MWD**: ≈  **Grip strength**: ≈  **Disability and functional capacity**: ≈ | **Enrolment**: 45 patients screened, of which n=22 were not eligible and n=8 declined to participate (n=4 not interested, n=2 conflicting timing with other life events, n=1 not committed to LuTx, n=1 safety monitor encouraged subject to decline participation).  **Attrition**: 13/15 (n=1 LuTx and n=1 did not initiate training despite multiple follow-up prompts).  **Acceptability**: Participants found the app interface intuitive and user-friendly. Participants found exercising at home convenient. The intervention was perceived as a rewarding experience by most of the participants.  **Fidelity** (participants): moderate training adherence: 60% of the prescribed exercise sessions were completed. Poor adherence to wearing the Fitbit device: 4/13 wore the device as prescribed.  **Fidelity** (interventionist): NA  **Safety**: No falls, injuries, or serious adverse events |
| **8** | Pehlivan, 2018a (Turkey) | LuTx candidates  Design: Prospective study  **I**: n=46  64% male  Age: 37 (±13) | **I:** At least 8 weeks, home-based, aerobic (supervised training: sets of 15 min cycling, walking, and arm ergometry, 5x/week, intensity: 60% of HRmax attained during 6MWT) + strength exercises (30% 1RM increasing according to patients’ tolerance, 2x/week group sessions supervised by physiotherapist) + breathing exercises (3x/week, unsupervised) | 6MWD  Dyspnea (MRC)  Knee extensors muscle strength  Elbow flexors muscle strength  FVC  FEV1  Depression (BDI)  QOL (SF-36)  **Assessments:**  - Before intervention  - After intervention | **6MWD:** ↑  **Dyspnea:** ↓ (improved)  **Knee extensors muscle strength:** ≈  **Elbow flexors muscle strength:** ≈  **FVC:** ≈  **FEV1:** ≈  **Depression:** ↓ (improved)  **QOL:** ↑ in subscales physical function and emotional role | **Enrolment**: n=46 assessed for eligibility and included  **Attrition**: 39/46 (85%) completed. Drop outs (n=7) due to LuTx  **Acceptability**: NR  **Fidelity** (participants): NR  **Fidelity** (interventionist): NR  **Safety**: NR |
| **9** | da Fontoura, 2018 (Brazil) | LuTx candidates (idiopathic pulmonary fibrosis)  Design: Retrospective pre-post cohort study  **I**: n=48  58% male  Age: 56.5 (±10.3) | **I:** 12-week, supervised aerobic and strength training (60 min/ session, 3x/week, intensity: Aerobic training 20 to 30 min of treadmill walking at a perceived effort of approximately 3/10; strength training 3 sets of 12 lightweight repetitions)  In addition nutritional, psychological, and disease ed­ucation management sessions 2x/month provided | 6MWD  QOL (SF36)  Dyspnea ( mMRC)  Impact of lung disease severity (FVC, DLCO, dyspnea, pulmonary artery systolic pressure) on the feasibility and response of the intervention  **Assessments:**  - Before intervention  - After intervention | **6MWD:** ↑  **SF36**: ↑ in sev­eral domains  **Dyspnea**: ↓ (improved)  **Lower limb** **effort perception** **↑** | **Enrolment**: n=48 assessed for eligibility and included  **Attrition**: 31/48 (65%) completed. Drop outs (n=17) due to death (n=6), non-compliance/ inability (n=11)  **Acceptability**: No differences in lung disease severity in patients completing vs. not completing the intervention; no association between disease severity and intervention effects.  **Fidelity** (participants): 11 patients did not complete PR due to noncompliance or inability; those who completed the intervention showed high attendance rate  **Fidelity** (interventionist): NR  **Safety**: NR |
| **10** | Ochman, 2018 (Poland) | LuTx candidates  Design: Non-randomized controlled trial  Total n=40  **I:** n=22:  100 % male  Age: 50.4 (±7.8)  **C:** n=18  89% male  Age: 53.6 (±8.8) | **I:** 12-weeks of Nordic walking**,** 2 cycles of 6 weeks. consisting of 2 weeks of rehabilitation under supervision of physiotherapists  during hospital stay and 4 weeks of homes based rehabilitation  Duration training sessions, frequency, intensity and supervision: NR  **C:**  No exercise | 6MWD  QOL (SF36)  Dyspnea (MRC, BDI)  Spirometry  **Assessments:**  - Before intervention  - After intervention | **6MWD**: I ↑ C red to comparator group  **SF36:** I ↑ C on physical component score  **Dyspnea**: I ↓ C based on MRC, but not on BDI)  **Spirometry**: I ≈ C | **Enrolment**: n=40 assessed for eligibility, of which app  **Attrition**: 40/40 (no dropouts)  **Acceptability**: NR  **Fidelity** (participants): NR  **Fidelity** (interventionist): NR  **Safety**: No adverse events observed |
| **11** | Byrd, 2019 (United States) | LuTx candidates  Design: Retrospective pre-post cohort study  **I:** n=141  60% male  Age: 58.5 (±14.9) | **I:** 1 month, supervised by physical therapists group-based, individualized ,aerobic, strength, balance, flexibility, and breathing exercises (ambulation on indoor track up to 20-30 min; cycling for 20 min; flexibility: 5-10 min; group exercise class: 30-minute, 5x/week, Intensity: Aerobic exercise: moderate intensity (4-6/10); strength exercise: 15-20 repetitions at intensity that induces muscle fatigue by end of set) + educational program | 6MWD  Dyspnea (SOBQ)  Depression (CES-D)  QOL (QLI)  **Assessments:**  - Before intervention  - After intervention | **6MWD:** moderate-to-large ↑  **Dyspnea**: ≈  **CES-D**: Small-to-moderate ↓  **QLI**: Small-to-moderate ↑ | **Enrolment**: n=153 assessed for eligibility, 141/153 included (92%; patients with pre and post-test 6MWD data)  **Attrition**: NR  **Acceptability**: NR  **Fidelity** (participants): NR  **Fidelity** (interventionist): NR  **Safety**: NR |
| **12** | Florian, 2019 (Brazil) | LuTx candidates  Design: Retrospective quasi-experimental case-control study  Total n=89  **I:** n=36  69% male  Age: 54.7 (±11)  **C:** n= 53  60% male  Age: 56.8 (±10.4) | **I:** 12-week, supervised by physical therapist aerobic, strength, breathing, and stretching exercises (~60 min/session, 3x/week, intensity: aerobic exercise: 70% 6MWT speed; strength exercise: 1 set of 10 repetitions at 30% 1RM) +  educational lectures, nutritional counselling, and social assistance  **C:** Patients who did not complete at least 36 exercise sessions (average: 10; range: 7-25) | 5-year survival Post-LuTx  6MWD  QOL (SF36)  Invasive mechanical ventilation  Length of stay at ICU  Length of hospital stay  **Assessments:**  - Before intervention  - After intervention | **5-year survival post-LuTx**: I ↑ C (90 vs. 61%)  **6MWD:** I ↑ C  **SF36:** I ↑ C on 4 domains: physical functioning, role physical, vitality, and role emotional  **Mechanical ventilation:** I ↓ C  **Length of stay at ICU**: I ↓ C (5 vs. 7 days)  **Length of hospital stay**: I ↓ C (20 vs. 25 days)  **Mortality during ICU**: I ↓ C | **Enrolment**: n=89 assessed for eligibility and included  **Attrition**: 36/89 (40%), reason for drop-out NR  **Acceptability**: NR  **Fidelity** (participants): 50% of patients did not complete the program  **Fidelity** (interventionist): NR  **Safety**: NR |
| **13** | McAdams-DeMarco, 2019 (United States) | KTx candidates (estimated to be within 3 to 6 months of KTx)  Design: Prospective cohort study with historic control group  Total n=49  **I:** n=24  61% male  Age: range 18-65+  **C:** n=25  40% male  Age: range 18-65+ | **I:** 2 months, supervised by physiotherapist assistant diaphragmatic breathing exercises, stretching, strengthening exercises, balance exercises, core stability, low impact cardiovascular exercises (40 min/session, 1x/week at outpatient clinic + daily at home, intensity NR)  **C:** No exercise | Feasibility: satisfaction with the intervention (scale 1 (very dissatisfied) to 5 (very satisfied)) and difficulty to implement intervention in daily life (1 (very difficult) to 5 (very easy))  Physical activity  Participant feedback  Length of hospital stay post-Tx  **Assessments:**  - Before intervention  - 1 month after intervention  - 2 months after intervention | **Physical activity**: ≈ at 1 month post-test within I; **↑** at 2 months post-test within I; comparison with C NR  **Length of stay**: I **↓** C (resp., 5 vs. 10 days) | **Enrolment**: 24/190 (13%) of eligible patients included. 87% of eligible patients declined to participate. Top 3 reasons: geographical limitations, too much commitment, transportation issues.  **Attrition**: 18/24 (75%), n=6 drop-outs (transportation issues, schedule conflicts, health/medical issues and too much of a time commitment)  **Acceptability**: 100% very satisfied with the prehabilitation program (score: 5.0); intervention was somewhat easy to implement in daily life (score: 4.1)  **Fidelity** (participants): NR  **Fidelity** (interventionist): NR  **Safety**: no safety concerns or deaths |
| **14** | Morkane, 2020 (UK) | LiTx candidates  Design: prospective cohort study, with non-randomized demographically matched control group  Total n=33  **I**: n=16  88% male  Age: 56 (±8)  **C**: n=17  82% male  Age: 56 (±8) | **I**: 6 weeks, supervised outpatient aerobic training on cycle ergometer. 3x/week, 40 min/session including interval training alternating moderate (80% of work rate at anaerobic threshold: 4-6x 3-min intervals) with severe (50% of the difference in work rates between work rate at anaerobic threshold and Wmax: 4-6x 2-min intervals) intensities.  **C**: Usual care (no exercise program)  Both groups received  standardized nutritional assessment and advice | VO_2_peak  Handgrip strength  Mid-arm muscle circumference  Post-LiTx ICU and hospital stay  6-months post-LiTx survival  **Assessments:**  - Before intervention  - Immediately after the intervention  - 6 weeks after the intervention | **VO_2_peak**: Immediately after the intervention: I ↑ and C ↓. 6 weeks after the intervention: no significant differences.  **Handgrip** **strength**: Immediately after the intervention: I ↑; C ≈. 6 weeks after the intervention: NR.  **Mid-arm** **muscle** **circumference**: I ≈, C ≈. 6 weeks after the intervention: NR.  **ICU** **stay**: I ≈ C  **Hospital** **stay**: Shorter in I compared to C (13 ± 6 vs. 30 ± 13 days, respectively)  **6-months post-LiTx survival**: I ≈ C | **Enrolment**: 33/61 of eligible patients included. I: n=9 declined, n=3 delisted/death, n=1 LiTx before start. C: n=7 declined, n=5 delisted/death, n=3 LiTx before approach).  **Attrition**: I: 9/16 completed the 6-week exercise intervention (n=4 deteriorated, n=2 LiTx, n=1 knee pain). C: 11/17 completed assessments at 6 weeks (n=3 LiTx, n=2 delisted, n=1 deteriorated). I: 8/16 completed follow-up assessment at 12 weeks (n=1 LiTx). C: 7/17 completed follow-up assessment at 12 weeks (n=2 LiTx, n=1 deteriorated, n=1 withdrew)  **Acceptability**: NR  **Fidelity** (participants): 127/135 exercise sessions (94%) completed by the 9 patients finishing the exercise intervention.  **Fidelity** (interventionist): NA  **Safety**: No adverse events related to exercise training. No incidents of worsening cirrhotic decompensation as a result of exercise. It was not reported whether knee pain in n=1, which led to drop out in I, was related to exercise or not. |
| **15** | Kılıç, 2020 (Turkey) | LuTx candidates  Design: Retrospective study  **I:** n=23  57% male  Age: 35 (±10) | **I:** 8-weeks, supervised outpatient clinic session and home-based, exercise training composed of aerobic (treadmill and cycle ergometer) + strength (resistance bands) + breathing exercises. Outpatient sessions: 60 min/session; 2x/week; Intensity aerobic exercise during outpatient sessions: 60% HRmax recorded during 6 min walking test; no other details reported  Home-based duration NR, 3x/week, intensity NR.  in addition psychological and nutritional support was provided | 6MWD  Dyspnea: Borg and MRC  **Assessments:**  - Before intervention  - After intervention | **6MWD**: **↑**  **Dyspnea**: **↓** (improved) | **Enrolment**: n=29 assessed for eligibility, 23/29 (79%) included exclusion due to LuTx n=4)  **Attrition**: 23/23 (100%)  **Acceptability**: NR  **Fidelity** (participants): NR  **Fidelity** (interventionist): NR  **Safety**: NR |
| **16** | Pehlivan, 2020 (Turkey) | LuTx candidates  Design: retrospective study  **I:** n=47  66% male  Age 39 (±15) | **I:** 12-week, group sessions, presumably supervised outpatient prehabilitation aerobic (treadmill and cycle and arm ergometry; 3 sets of 15 min, intensity HR_rest_ + 60-80% HR_reserve_) + strength (free weights; 1-2 sets of 8-12 repetitions of 5 major muscle groups, intensity 20-40% 1 RM) exercises  Frequency: NR | 6MWD  Knee extensor muscle strength  Handgrip strength  MIP  MEP  FEV1  FVC  Dyspnea (MRC)  **Assessments:**  - Before intervention  - After intervention | **6MWD:** ↑  **Knee extensor muscle strength:** ≈  **Handgrip strength:** ↑  **MIP:** ↑  **MEP:** ↑  **FEV1:** ≈  **FVC:** ≈  **Dyspnea:** ↓ (improved) | **Enrolment**: NR  **Attrition**: NR  **Acceptability**: NR  **Fidelity** (participants): NR  **Fidelity** (interventionist): NR  **Safety**: NR |
| **17** | Lorenz, 2020 (United States) | KTx candidates  Design: Prospective cohort study  **I:** n =27  57% male  Age: 62 (IQR 53-67) | **I:** 8-week**,** supervised by respiratory therapist, 2x/week, 60 min/session, aerobic training, (treadmill walking or hand pedal ergometry, duration progressing to 10 min of continuous training, intensity: moderate to high intensity (>60% of maximal work rate), adjusted weekly based on target Borg dyspnea or fatigue scores of 4-6) + strength training (resistance bands, duration NR, 2x/week, intensity: started at 60% 1RM; 1 to 3 sets of 8-12 repetitions targeting upper and lower limbs muscles) + flexibility training. | Frailty (Fried frailty phenotype, FFP parameters)  Body composition (BIA)  SPPB: balance, chair stands, and gait speed  QOL (KDQOL-SF)  **Assessments:**  - Baseline  - Halfway point (4 weeks)  - After intervention | **Frailty FFP:** ≈  **Frailty parameters**:  - Physical activity ↑  - Walking speed ↑  - Grip strength ↑  **Body composition:** ≈ subgroup of participants with baseline wasting experienced ↓ in SMI and ASMI compared to those without baseline wasting  **SPPB:** ↑  **SPPBparameters:**  - Balance ↑  - Chair stands ↑  - Gait speed ↑  **KDQOL:** ≈ | **Enrolment**: n=29 assessed for eligibility, 27/29 (93%) included  **Attrition**: 19/27 (70%) drop-outs due to health problems (n=5), lack of time (n=1), lack of transportation (n=1), and other (n=1).  **Acceptability**: 100%, 90% of respondents reported that the intervention was beneficial to their overall (physical and mental) health, that they felt more confident about exercise and planned to continue the exercise intervention  **Fidelity** (participants): 91% of the participants that did not dropout completed all 16 scheduled exercise sessions  **Fidelity** (interventionist): NR  **Safety**: no study-related adverse events occurred |
| **18** | Massierer, 2020 (Canada) | LuTx candidates  Design: Pre-post retrospective study  **I:** n=159  57% male  Age: 49 (±14) | **I:** During the waiting list period, unsupervised, home-based aerobic exercises (stationary cycling, treadmill/indoor walking, stair climbing, duration sessions progressed from 5-10 min to 30 min 5x/week, intensity: 3-4/10 dyspnea and Borg score) + strength exercises (Elastic bands, free weights, and body weight exercises, 2-3 sets of 10 repetitions of each muscle group; 3x/week, intensity: ≥5/10 Borg score) + flexibility exercises (Upper and lower limbs stretching, 20-30 s stretch per muscle group, daily) | 6MWD  **Assessments:**  - Assessment for LuTx  - Last test prior to LuTx  - 1-month post-LuTx | **6MWD**: ↓ (28 ± 94 m) between assessment LuTx and last test prior to LuTx | **Enrolment**: n=212 assessed for eligibility; 159/212 (75%) included  **Attrition**: 159/159 (100%) completed  **Acceptability**: NR  **Fidelity** (participants): 85% of the patients reported that they had done the exercises at home during the waiting time list (assessed by 1 question)  **Fidelity** (interventionist): NR  **Safety**: no adverse events |
| **19** | Wickerson, 2020 (Canada) | LuTx candidates  Design: Retrospective study  **I:** n=62  37% male  Age: 62 (IQR 56-67) | **I:** 6-week, supervised, hospital-based, outpatient prehabilitation comprised of aerobic (treadmill and cycle ergometer, moderate intensity) + strength (functional and resistance training) + stretching exercises (90 min/ session, 3x/week) | SPPB:  - 5x STS  - Balance  - Gait speed  6MWD  **Assessments:** | **SPPB**: **↑**;  - 5xSTST: **↑**  - Balance: ≈  - Gait speed: ≈  **6MWD**: ≈ | **Enrolment**: n=253 assessed for eligibility. 150/253 (59%) included (accepted for listing LuTx)  **Attrition**: 62/150 (41%) completed intervention and had follow-up data. Dropouts due to delisting or death (n=15), LuTx (n=57), no follow-up data (n=15), prehabilitation put on hold (n=1)  **Acceptability**: NR  **Fidelity** (participants): NR  **Fidelity** (interventionist): NR  **Safety**: NR |
| **20** | Lin, 2021 (United States) | LiTx candidates  Design: Ambispective cohort study  I n=305  59% male  Age: 61 (53-66) | **I:** individualized home-based exercise plan with in-person physiotherapy visits every 1-3 months . Robust patients: encouraged to remain active and continue previous exercise regimens;  (pre-) frail patients: specific home-based exercise workouts focusing on upper and lower body muscles using weights and resistance bands (30 min/session, 5x/week, 10-30 repetitions per exercise, inducing a subjective challenge for the patient + promotion of incorporation of aerobic exercise | Frailty (Liver Frailty Index)  6MWD  Gait speed  Survival (1-year): associations of exercise plan with frailty and survival  **Assessments:** Every 1-3 months at in-person appointment with physiotherapist (actual range 90-120 days) | **Liver Frailty Index**: ↑  **6MWD**: ↑  **Gait speed**: ≈  **Survival 1-year:** positive association with number of attended physiotherapy visits; no association with self-reported adherence to exercise plan; negative association with baseline frailty; positive association with improvements in frailty | **Enrolment**: n=305 assessed for eligibility  **Attrition**: 211/305 (69%); dropouts due to non-adherence(n=94)  **Acceptability**: NR  **Fidelity** (participants): 30.8% did not attend any physiotherapy appointment  **Fidelity** (interventionist): NR  **Safety**: NR |
| **21** | Kerti, 2021 (Hungary) | LuTx candidates  Design: Single-arm observational study  **I**: n=63  54% male  Age: 58 (±7) | **I**: 4-week,  - Type: aerobic (continuous or interval cycling or walking/running) and breathing exercises  - Duration training sessions: aerobic: 15-20 min  - Frequency: aerobic: 2-3x/day  - Intensity: dyspnea and leg fatigue Borg score of 7/10  - Supervision: supervised | 6MWD  Handgrip strength  Lung volumes and function (FVC, IVC, FEV1)  MIP  Flexibility (chest wall expansion)  Breath holding test  QOL (CAT)  Dyspnea (mMRC)  COPD severity (BODE-index)  **Assessments:**  - Before intervention   - After intervention | **6MWD: ↑**  **Handgrip strength**: ≈  **FVC**: ≈  **IVC:** ≈  **FEV1**: ≈  **MIP**: ≈  **Cchest wall expansion:** ↑  **Breath holding test**: ≈  **CAT:** ↑  **mMRC**: ≈  **BODE-index**: ≈ | **Enrolment**: NR  **Attrition**: NR  **Acceptability**: NR  **Fidelity** (participants): NR  **Fidelity** (interventionist): NR  **Safety**: No cardiovascular or other side effects were detected |
| **22** | Layton, 2021 (United States) | LuTx candidates  Design: Prospective study, with historic control group  Total: n=19  **I:** n=11  54% male  Age: 30 (IQR: 10)  **C:** n=8  0% male  Age 29 (IQR 7) | **I:** 12-week (24 sessions)**,** semi-supervised home-based aerobic, strength, and stretching exercise program, administered via a fitness application with self-selected activities, exercise videos, and monitoring of exercise time and heart rate (10 min strength and 20 min for cycling training, 2x/week, intensity: personalized heart rate targets and Borg scale score). Study team members send weekly personalized exercise plans by email and reviewed the exercise logs on a weekly basis  **C:** Retrospective dataset of 8 cystic fibrosis patients who participated in a 24-session outpatient hospital-based rehabilitation program | Adherence (number of sessions completed in 12 weeks)  6MWD  Dyspnea  Adverse events  **Assessments:**  - Before intervention  - After intervention | **Adherence**: 5/11 in the intervention group compared to 0/8 in the comparator group completed ≥24 sessions in 12 weeks  **6MWD**: ≈ in those completing ≥24 sessions; ↓ in those not completing ≥24 sessions  **Dyspnea**: ≈ | **Enrolment**: n=18 assessed for eligibility to participate in intervention group, 11/18 (61%) included in intervention group  **Attrition**: 11/11 (100%) in intervention group  **Acceptability**: 5/18 patients approached to participate in the intervention group were not interested in participating; 3/11 participants of the intervention group purchased the application upon study completion and continued using it on their own; history of physical activity correlated with intervention adherence  **Fidelity** (participants): better adherence in the intervention group compared to comparator group (19 ± 12 vs. 9 ± 4 completed sessions)  **Fidelity** (interventionist): NR  **Safety**: No serious adverse events observed. Most frequently reported symptoms with exercise: muscle fatigue (3/11), cough or wheezing (3/11), dyspnea (1/11), and transient headache after exercise (2/11). Also reports of chest discomfort during exercise due to acute infection (n=1), light-headedness during exercise due to low blood sugar (n=2), and haemoptysis during push-ups (n=1) |
| **23** | Wickerson, 2021 (Canada) | LuTx candidates  Design: Prospective  **I:** n=84  47% male  Age: 59 (±12) | **Intervention group:**  4-week, home-based, telehealth support by the physiotherapist (phone, video, texting, and remote monitoring) web-based monitoring app platform for prehabilitation with individually tailored aerobic and strength exercises (duration per session NR, 3x/week, intensity NR) | Evaluation of the web-based app (app usage, satisfaction with the app)  Physical activity (RAPA)  Self-efficacy for exercise  Training volume  6MWD  SPPB  Exertional O_2_ requirement  **Assessments:**  - Before intervention  - After intervention | **Physical activity:** ↑  **Self-efficacy for exercise**: Increased (n=17), decreased (n=5), or remained the same (n=15).  **Training volume:** ↑ walking  **6MWD:** ↓  **SPPB:** ≈  **Exertional O_2_ requirement:** ↑ | **Enrolment**: n=84 assessed for eligibility  **Attrition**: satisfaction survey 42/84 (50%), physical activity survey 23/84 (27%), exercise self-efficacy survey 37/84 (44%), exercise volume 78/84 (88%), (6MWD 45/84 (54%), and SPPB 42/84 (50%) Dropouts: reasons for missing data NR  **Acceptability**: patients: 78/84 patients used the app for at least 4 weeks. Reasons for not using the app: no smartphone or tablet (n=1), no cellular data alongside limited Wi-Fi (n=2), declined (n=1), inpatient (n=1), LuTx (n=1); 88% liked the virtual care features of the app; 83% agreed that it helped to prepare them for LuTx.  Physiotherapists (n=3): overall satisfaction using the app to maintain communication, provide virtual support, and remotely monitor patients during the COVID-19 pandemic; not fully confident conducting remote clinical assessments using the app or identifying an early clinical change; prefer to bring patients on site for functional or exertional O_2_ reassessment when possible; patient access to equipment and monitoring devices and lack of integration with Bluetooth devices for biometrics reported as strong barriers by the physiotherapists  **Fidelity** (participants): 64% entered ≥10 prescribed exercise sessions into the app during the study time frame  **Fidelity** (interventionist): NR  **Safety**: no serious adverse events |
| **24** | Duarte-Rojo, 2021 (United States) | LiTx candidates  Design: prospective, single group, pre-post study, mixed methods  I n=25  57% male  Age 60 (±8) | **I:** 6-week, unsupervised, home-based training guided by a smartphone application (EL-FIT) and tracker comprised of strength and mobility training, low-intensity training, moderate-intensity training, balance training, and miscellaneous. Duration per session and frequency: Variable, based on participant choice. Intensity of exercise training levels was allocated by stratification algorithm | Ability of the EL-FIT algorithm to correctly stratify patients into the level of training intensity (compared to a clinician – LiTx physical therapist)  Usability of the app  Daily steps  **Assessments:**  - Baseline,  - Week 1-6 | **EL-FIT app:** correctly prescribed level of training in 89% of cases; no difference between the app and the clinician  Training level prescription was not associated with the presence of frailty for either the EL-FIT app or by the clinician or physical therapists  **Usability**: 77% of participants watched at least 1 video; 69% completed 1 section of videos (either exercise or education)  **Daily steps:** decreased in 30% of participants, stayed the same in 35%, and increased in 35% | **Enrolment**: n=28 assessed for eligibility  **Attrition**: 25/28 (89%) completed; Dropouts due to: stay in clinic too short to get the apps downloaded (n=2), LiTx (n=1)  **Acceptability**: NR  **Fidelity** (participants): NR  **Fidelity** (interventionist): NR  **Safety**: NR |
| **25** | Byrd, 2022 (United States) | LuTx candidates  Design: Prospective pre-post cohort study  I n=57  44% male  Age 50 (±16) | **I:** 1 month, supervised by physical therapists group-based; individualized aerobic, strength, balance, flexibility, breathing exercises and educational program (duration training sessions: ambulation on indoor track up to 20-30 min; cycling for 20 min continuously; flexibility: 5-10 min; group exercise class: 30-minute, 5x/week, intensity: Aerobic exercise: moderate intensity (4-6/10); strength exercise: 15-20 repetitions at intensity that induces muscle fatigue by end of set) | Balance assessed (FAB/ SF-FAB )  Balance and coordination (FSST)  6MWD  5x STST  Gait speed  Balance: Limits of Stability Test  **Assessments:**  - Before intervention  - After intervention | **FAB**: ↑ (moderate change)  **SF-FAB**: ↑ (moderate change)  **FSST**: ↑ (moderate change)  **6MWD**: ↑ (large change)  **5x STS**: ↑ (moderate change)  **Gait speed**: ↑ (small change)  **Limits of Stability Test**: ↑ (moderate change) | **Enrolment**: n=79 assessed for eligibility, 57/79 (72%) included  **Attrition**: 39/57 (68%) of included, drop-out reasons NR  **Acceptability**: 38% declined to participate  **Fidelity** (participants): 3.5% unable to complete post-training assessment  **Fidelity** (interventionist): NR  **Safety**: 2 reported falls during the rehabilitation program |
| **Psychosocial interventions** | | | | | | |
| **1** | Craig, 2016  (Canada) | Liver Tx candidates  Kidney Tx candidates  N=41  **I** n=41  Kidney Tx: n=16  38% male  Age 49.5 (19-65)  Liver Tx n=25  56% male  Age 56 (22-64) | **I**: 8-weeks, 2-hour/week, group-based, coping skills training utilizing elements of CBT, narrative, and mindfulness, delivered by a social worker | Anxiety (HAM-A)  Depression (HAM-D)  Coping (brief COPE)  **Assessments:**  - Before intervention  - After intervention  - Follow-up 1 month after intervention | **HAM-A**: I ↓ score from pre-post intervention, but not at follow-up  **HAM-D**: I ↓ score from pre-post intervention, but not at follow-up  **COPE**: I ↓ score on subscales denial, self-blame, and instrumental support from pre to post intervention; I ↑ score on subscales religion and acceptance and cluster scale emotion focused coping from pre to post intervention; I ↑ score on subscales acceptance and instrumental support remained at 1-month follow-up;  I ≈ C on cluster scale scores problem-focused coping or dysfunctional coping | **Enrolment:** NR  **Attrition**: 37/41 (90%)  **Fidelity (participants)**: 1 participant missed 3 group sessions; no other participant missed more than 2 sessions  **Fidelity (interventionist):** NR  Acceptability: helpful to manage symptoms of anxiety and depression  **Safety:** one participant started medication treatment during the intervention |
| **2** | Febrero, 2019 (Spain) | Liver Tx candidates  N = 25  **I** n=15  87% male  Age 55 (49-63)  **C** n=10  80% male  Age 55 (43-64) | **I**: 6-months, 12 sessions, 2.5-hour/ fortnightly, group-based, in person, psychotherapy and coping skills training delivered by a psychologists and psychotherapist  **C**: no intervention | Depression (BDI)  **Assessments:**  - Before intervention  - After intervention | **BDI:** I ↓ score after study completion; C ↑ score at 6 months after baseline | **Enrolment**: NR  **Attrition**: I: 7/15 (47%) & C 5/10 (50%) completed all assessments (n= 5 transplant; n=5 low attendance rate; n=2 medical problems)  **Fidelity (participants)**: I n= 5 attended <50% of sessions  **Fidelity (interventionist**): NR  **Acceptability**: NR  **Safety:** NR |
| **3** | Jutagir, 2019  (USA) | Liver Tx candidates  N = 29  **I** n=29  72% male  Age 55.1 (±7.2) | **I**: 8-weeks, 1 hour/ week, group-based, in person CBT stress management and relaxation training, delivered by a psychologist | Anxiety (BAI)  Depression (BDI)  **Assessments:**  - Before intervention  - After intervention | **BAI**: ≈ in symptom level  **BDI**: ≈ in symptom level | **Enrolment:** 29/120 (24%), 4/29 dropped out (14%)  **Attrition**: 17/25 completed postintervention survey (68%)  **Fidelity (participants):** 5.6 of 8 sessions, 69% attendance rate  **Fidelity (interventionist):** NR  **Acceptability:** intervention rated as good or excellent by participants and helped them to feel supported  **Safety:** No adverse events |
| **4** | Zhao, 2021  (China) | Kidney Tx candidates (on dialysis)  N = 37  **I** n=18  78% male  Age 35.3 (±6.7)  **C** n=19  72% male  Age 40.4 (±12.0) | **I**: 1 month, 0.5 hour/week, group-based, dedicated psychological counselling delivered by trained nurses  **C**: standard nursing care, historic cohort | Psychological status (MSSNS)  Subscales MSSNS:  - anxiety  - depression  - anger  - loneliness  **Assessments**:  1 month prior to Tx  Day before Tx | **MSSNS**: I ↓ total score and ↓ score on all subscale than C | **Enrolment**: NR  **Attrition**: NR  **Fidelity (participants):** NR  **Fidelity (interventionist):** NR  **Acceptability:** NR  **Safety**: NR |

*Abbreviations:*

Tx: transplant, I: intervention group, C: comparator group, NR: Not reported, CAT: COPD Assessment Test; NR: not reported; mMRC: modified Medical Research Council Dyspnea Scale; ECMO: Extra Corporal Membrane Oxygenation; LuTx: Lung transplant; LiTx: Liver transplant; KTx; Kidney transplant; MRC: Medical Research Council scale; BDI: Beck Depression Inventory; STAI: State-Trait Anxiety Inventory; SOBQ: San Diego Shortness of Breath Questionnaire; CESD: Center for Epidemiological Studies-Depression Scale ;QLI: Quality of Life Index; LFI: Liver Frailty Index; FAB: Fullerton Advanced Balance Scale; SF-FAB: short-form Fullerton Advanced Balance Scale; FSST: Four Square Step Test; RAPA: Rapid Assessment of Physical Activity questionnaire; CBT: cognitive behavioural therapy, HAM-A: Hamilton Anxiety Rating Scale, HAM-D: Hamilton Depression Rating Scale, BDI: Beck Depression Inventory; BAI: Beck Anxiety Inventory; Brief Cope: Coping questionnaire and the MSSNS: The Mental Status Scale in Non-Psychiatric Settings.
